# Making and Breaking Supramolecular Synthons for Modular Protein Frameworks

**DOI:** 10.1002/chem.202500732

**Published:** 2025-04-16

**Authors:** Niamh M. Mockler, Colin L. Raston, Peter B. Crowley

**Affiliations:** ^1^ School of Biological and Chemical Sciences University of Galway University Road Galway H91 TK33 Ireland; ^2^ Flinders Institute for Nanoscale Science and Technology College of Science and Engineering Flinders University Bedford Park SA Adelaide 5042 Australia

**Keywords:** affinity tag, crystal engineering, macrocycle, recognition, self‐assembly

## Abstract

Anionic calixarenes are useful mediators of protein assembly. In some cases, protein – calixarene cocrystallization yields multiple polymorphs. *Ralstonia solanacearum* lectin (RSL) cocrystallizes with *p*‐sulfonato‐calix[8]arene (**sclx_8_**) in at least four distinct pH‐dependent arrangements. One of these polymorphs, occurring at pH ≤ 4, is a cubic framework in which RSL nodes are connected by **sclx_8_** dimers. These dimers are supramolecular synthons that occur in distinct crystal structures. Now, we show that the discus‐shaped dimer of *p*‐phosphonato‐calix[6]arene (**pclx_6_**), can replace the **sclx_8_** dimer yielding a new assembly of RSL. Remarkably, just one type of RSL – **pclx_6_** cocrystal was formed, irrespective of pH or crystallization condition. These results with **pclx_6_** contrast starkly with **sclx_8_** and suggest that the calixarene type (e.g., phosphonate versus sulfonate) dictates the synthon durability, which in turn exerts control over protein assembly and polymorph selection. *Breaking* the **pclx_6_** dimer required a mutant of RSL with an affinity tag for macrocycle binding. This highly accessible, dicationic site resulted in a significantly altered and porous framework with **pclx_6_** (but not with **sclx_8_**). Experiments with ternary mixtures of RSL, **pclx_6_**, and **sclx_8_** provide evidence of pH‐driven self‐sorting. Thus, the “mix‐and‐match” of protein and supramolecular synthons is a promising approach to protein crystal engineering.

## Graphical abstract

Calixarenes are useful tools for protein crystal engineering. With the model protein RSL, different calixarene dimers generate distinct frameworks. An N‐terminal macrocycle binding tag serves to expand the structural landscape.

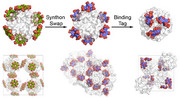

## 1 Introduction

There is great interest in the modular construction of nanoporous crystals by using interchangeable building blocks.^[^ ^1^, ^2^, ^3^, ^4^, ^5^, ^6^, ^7^, ^8^, ^9^, ^10^ ^]^ A series of isoreticular metal–organic frameworks (MOFs) can be generated by substituting the organic linkers with equivalent units of varying dimensions, yielding materials of controlled porosity.^[^ ^2^ ^]^ Likewise, porous organic cage (POC) cocrystals with tunable properties can be assembled from the modular “mix‐and‐match” of prefabricated organic cages.^[^ ^1^ ^]^ Protein‐based frameworks are sustainable, biocompatible alternatives to MOFs and POCs, with the additional benefit of intrinsic functionality (e.g., enzymatic activity, selective ligand binding). However, rational protein crystallization is challenging due to surface heterogeneity, with multiple weak interactions contributing to protein – protein interfaces. Advances in protein crystal engineering have been driven by *de novo* design,^[^ ^4^, ^10^ ^]^ protein engineering^[^ ^5^ ^],^ and metal‐ or ligand‐mediated assembly.^[^ ^3^, ^6^, ^7^, ^8^, ^9^ ^]^ The latter approach includes macrocycles with predictable protein recognition properties.^[^ ^8^, ^11^, ^12^, ^13^, ^14^, ^15^, ^16^, ^17^, ^18^, ^19^, ^20^, ^21^ ^]^ Symmetric and chemically uniform, the macrocycle acts as *molecular glue* by masking the protein, simplifying the surface features, and providing a homogenous scaffold for controlled oligomerization and crystallization. While the database of protein – macrocycle frameworks is limited in size, the possibility of using supramolecular synthons^[^ ^22^ ^]^ in protein crystal design is immanent.^[^ ^19^ ^]^ Reproducible protein – calixarene and calixarene – calixarene interfaces have been identified across multiple distinct cocrystal structures.^[^ ^8^, ^13^, ^15^, ^17^, ^18^, ^19^ ^]^ However, the rational application of these synthons, i.e., deliberate incorporation into new structures, is underexplored.

In this work, we report modular protein – calixarene cocrystallization. Previously, we showed that the anionic, water‐soluble *p*‐sulfonato‐calix[8]arene (**sclx_8_**) yields three or four polymorphs with the model proteins cytochrome *c* (p*I* ≈9.5)^[^ ^15^ ^]^ and trimeric *Ralstonia solanacearum* lectin (RSL, p*I* ≈6.5),^[^ ^8^, ^18^, ^19^ ^]^ respectively. In each model system, polymorph selection depends on the crystallization condition (pH and ionic strength). The variety of protein – **sclx_8_** polymorphs is partly attributable to the macrocycle flexibility, enabling molding to the protein surface and the formation of distinct interaction patches. A special case of these patches arises when the macrocycle forms oligomers.^[^ ^19^ ^]^

**Figure 1:**
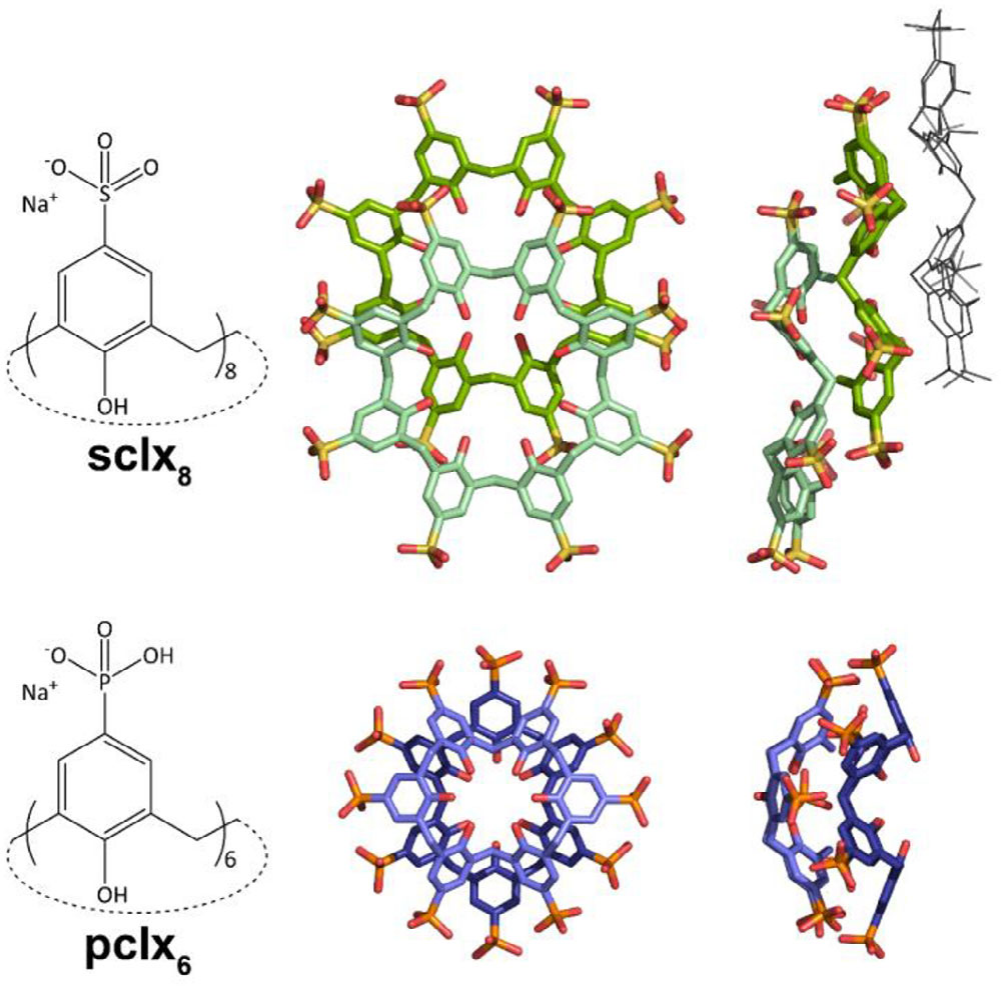
**Calixarene oligomers. sclx_8_** adopts the *C*_4_‐symmetric pleated loop conformation and assembles in *staggered* stacks via CH−*π*, OH−*π*, *π–π*, and anion−*π* interactions.^[^ ^8^, ^19^ ^]^ **pclx_6_** adopts the *C*_2_‐symmetric double‐cone conformation and dimerizes *face‐on* via CH−*π* and *π–π* interactions. In addition, intermolecular hydrogen bonding occurs at the phosphonates^.[^ ^13^, ^17^ ^]^ While **sclx_8_** can form extended oligomers (trimer indicated in wireframe), **pclx_6_** forms discrete dimers.

The **sclx_8_** – **sclx_8_** structural unit (Figure 1) is a supramolecular synthon that occurs in two distinct protein – calixarene frameworks and in the sodium – **sclx_8_** salt, with the pleated **sclx_8_** capable of stacking into dimers, trimers, and higher order oligomers.^[^ ^8^, ^19^ ^]^ A porous RSL – **sclx_8_** cubic cocrystal (PDB 6z5 g, space group *I*23, 66% solvent content) is directed by these staggered calixarene dimers linking the protein nodes. This cocrystal only grows at pH ≤ 4, which makes the protein cationic as well as favoring macrocycle oligomerization. Mediated entirely by calixarene – calixarene and protein – calixarene interfaces, the RSL – **sclx_8_** cubic cocrystal^[^ ^8^ ^]^ serves as a template for “mix‐and‐match” assembly. We were motivated to test if this framework could be reconstituted by replacing the macrocycle with a different dimer‐forming type.

*p*‐Phosphonato‐calix[6]arene (**pclx_6_**) forms a discus‐shaped, discrete^[^ ^23^ ^]^ dimer with a hydrogen‐bonded phosphonate rim (Figure 1). This **pclx_6_** – **pclx_6_** synthon mediates two related assemblies of cytochrome *c*.^[^ ^13^, ^17^ ^]^ Now, we apply **pclx_6_** to RSL assembly. Surprisingly, and despite exhaustive searching, we obtained only one type of RSL – **pclx_6_** cocrystal. The **pclx_6_** dimer proves to be a durable synthon, unaffected by pH, and facilitating a single packing mode. Using simple models, we demonstrate that **pclx_6_** binding to RSL is predictable based on the RSL – **sclx_8_** interfaces,^[^ ^8^ ^]^ and we provide pointers for the predictability of crystal packing.^[^ ^8^, ^13^, ^17^, ^19^ ^]^ Expanding the RSL – **pclx_6_** crystal engineering landscape was made possible by protein engineering. The variant MK‐RSL has a sterically accessible N‐terminal methionine‐lysine motif.^[^ ^16^, ^21^ ^]^ This binding tag engaged **pclx_6_** via a different synthon, generating a new framework. Together, these results suggest the application of calixarenes in modular protein crystal engineering.

## 2 Results and Discussion

### 2.1 Mix‐and‐Match Protein – Calixarene Assembly

**Figure 2:**
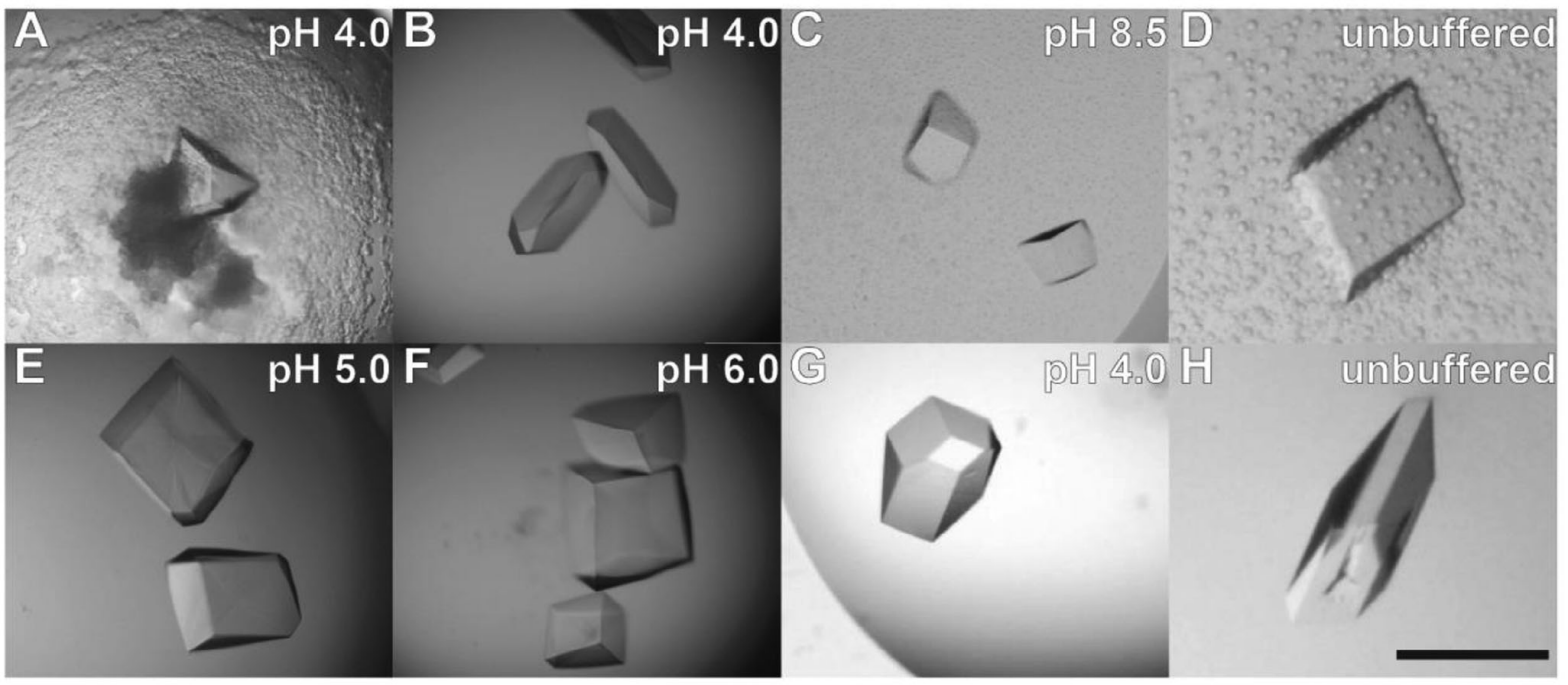
RSL – **pclx_6_** cocrystals of diverse morphology were obtained in a wide pH range using ammonium sulfate A–D), sodium citrate E,F), or PEG 3350 G,H) as the precipitant. The scale bar is 200 µm. All conditions yielded the same crystal form in space group *H*32.

Cocrystallization trials of RSL and **pclx_6_** were performed with the aim of reconstituting the cubic (*I*23) RSL – **sclx_8_** scaffold.^[^ ^8^ ^]^ We hypothesized that the **sclx_8_** dimer could be replaced by an alternative supramolecular synthon, the **pclx_6_** dimer, generating a new framework with altered properties. RSL – **pclx_6_** mixtures were extensively screened using a commercial screen (JCSG++ HTS, Jena Biosciences) and in‐house conditions. Cocrystals of 100–200 µm dimension and diverse morphology grew within hours to days in a broad range of conditions (Table 1 and Figure 2). Interestingly, some of these conditions were the same as those that yield RSL – **sclx_8_** polymorphs (Table 1). Note that trials with RSL and *p*‐phosphonato‐calix[8]arene (**pclx_8_**) or *p*‐sulfonato‐calix[6]arene (**sclx_6_**) did not yield cocrystals.

**Table 1:** RSL – **calixarene** cocrystallization conditions and crystal structure properties.^*^

Precipitant	Buffer [0.1 m]	pH	Salt [0.2 m]	Cocrystal Form [% Solvent Content^[^ ^a^ ^]^	
				RSL – sclx_8_	RSL – pclx_6_
20% PEG 3350	–	–	NaSCN	–	*H*32 (44)
20% PEG 3350	–	–	KNO_3_	–	
20% PEG 8000	K/PO_4_^2−^/citrate	4.2	NaCl	–	
1% PEG 3350 / 1 m (NH_4_)_2_SO_4_	Bis‐Tris	5.5–6.0	–	–	
18% PEG 3350	Na citrate	4.0	–	–	
0.4–1.2 m (NH_4_)_2_SO_4_	Na citrate	4.0–6.0	–	*I*23 (66)	
0.6–1.0 m Na citrate	–	5.0–6.0	–	*H*32 (51)	
–	Na acetate	4.0	NaCl	*P*3 (59)	
1.0–1.25 m (NH_4_)_2_SO_4_	Tris‐HCl	8.5	(Li)_2_SO_4_	*P*2_1_3 (36)	

* RSL – **sclx_8_** data taken from references 8 and 18.

^[a]^ Solvent content estimated from total mass (protein plus calixarene).

We tested numerous different RSL – **pclx_6_** cocrystals at SOLEIL synchrotron on four separate occasions, collecting data to 1.1 Å resolution (Table S1). Irrespective of the precipitant or the crystallization pH, all crystals were the same in the trigonal space group *H*32. As hypothesized, the dimeric **pclx_6_** – **pclx_6_** synthon^[^ ^13^, ^17^ ^]^ directs protein assembly.

**Figure 3:**
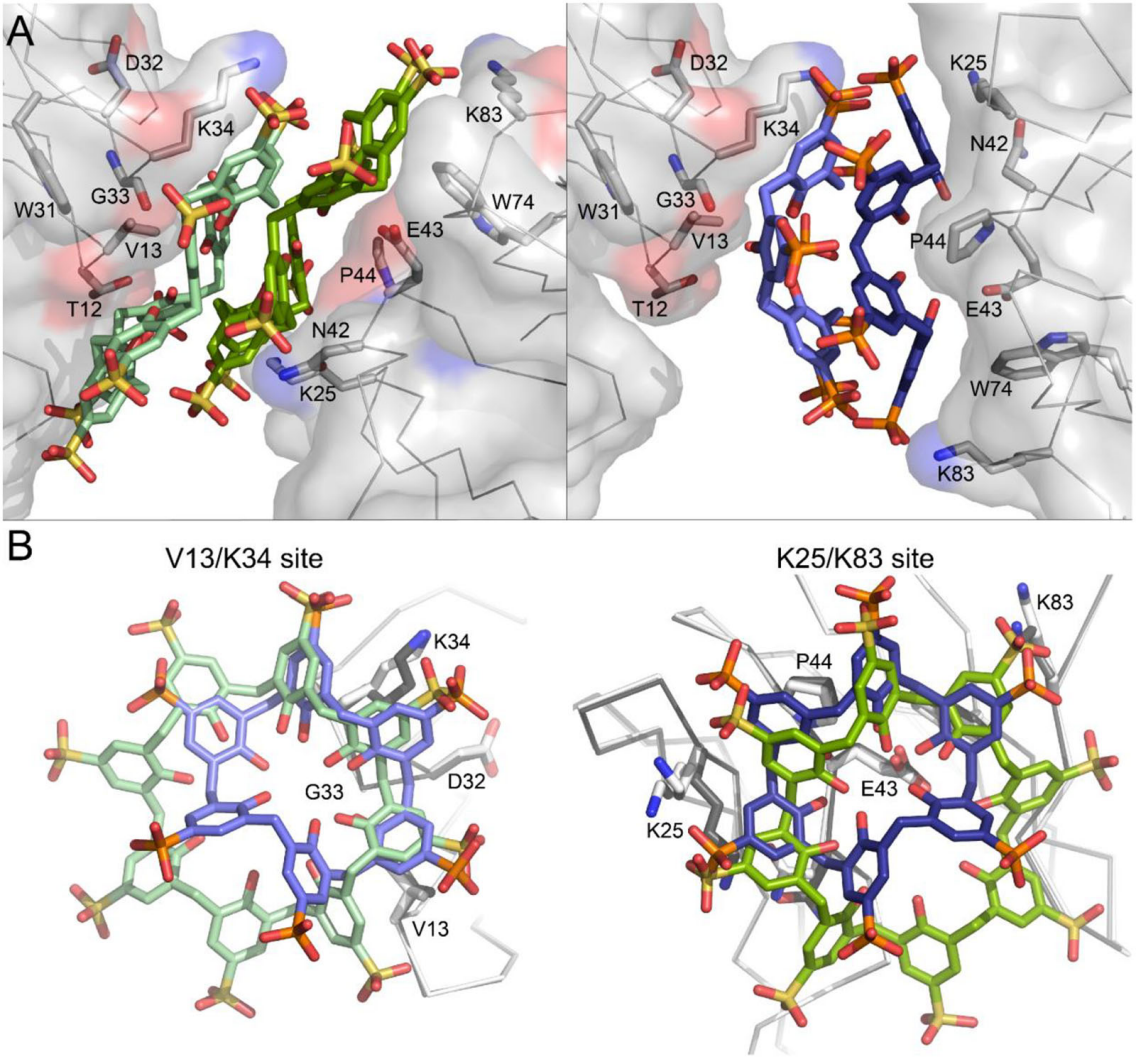
**Mix‐and‐match assembly**. A) Two RSL trimers (nodes) assembled on dimeric calixarene linkers, **sclx_8_** or **pclx_6_**. B) Superposition of the RSL – **pclx_6_** and the RSL – **sclx_8_** binding sites reveals approximate overlap of 3 and 4 calixarene units at the Val13/Lys34 and Lys25/Lys83 sites, respectively. The calixarenes are shades of green (**sclx_8_**) or blue (**pclx_6_**) and the RSL proteins are light (**sclx_8_** structure) or dark (**pclx_6_** structure) grey.

The RSL – **pclx_6_** *H*32 and the RSL – **sclx_8_** *I*23 structures are analogous, each mediated by a calixarene dimer linking protein nodes via two distinct surface patches; the Lys25/Lys83 and Val13/Lys34 sites (Figure 3). This synthon substitution, replacing the **sclx_8_** dimer with the **pclx_6_** dimer, is an example of a “mix‐and‐match” assembly^[^ ^1^ ^]^ and results in a framework that is accessible across a wide range of conditions. While the cubic RSL – **sclx_8_** cocrystals grow only at pH ≤ 4 (favoring **sclx_8_** dimerization),^[^ ^8^, ^19^ ^]^ the trigonal RSL – **pclx_6_** cocrystals grow at pH 4–9, in the presence or absence of a precipitant (Table 1). Apparently, the **pclx_6_** dimer is stable in a wider range of conditions than the **sclx_8_** dimer, possibly due to differences in the acidic *para* substituent.^[^ ^24^, ^25^ ^]^ In the pH range tested, **pclx_6_** likely has a formal charge of ‐6 and face‐on dimerization is enabled by hydrogen bonding between the singly deprotonated phosphonate groups.^[^ ^24^, ^25^, ^26^, ^27^ ^]^ Such stabilizing interactions are not possible in **sclx_n_** dimers with monoprotic sulfonates. Although the **pclx_6_** and **sclx_8_** dimers are interchangeable in RSL assembly, attempts to cocrystallize **sclx_8_** dimers with cytochrome *c* were unsuccessful (see methods). Cocrystallization of **sclx_8_** and cytochrome *c* at pH 4 was impeded by heavy precipitation. As the **pclx_6_** dimer is accessible in a wide range of pH, it may be preferable for assembling cationic proteins.^[^ ^13^, ^17^ ^]^

Synthon substitution with RSL works, despite differences in synthon size, shape, symmetry and functionality, as the **pclx_6_** dimer and **sclx_8_** dimer bind to similar patches on the protein surface (Figure 3). The larger RSL – **pclx_6_** interface (270 Å^2^) buries Asn42, Glu43, Pro44 and Trp74. The Glu43 side‐chain hydrogen bonds to a phenol of **pclx_6_**, while Pro44 forms CH−*π* interactions with a pair of phenolphosphonates. This interface core is flanked by Lys25 and Lys83, which form salt bridges to the phosphonates. The smaller RSL – **pclx_6_** interface (200 Å^2^) buries Val13, Gly33 and Lys34. The Gly33 carbonyl group is within hydrogen bond distance of four phenols, while Val13 forms CH−*π* interactions with a single phenolphosphonate. Lys34 is a linchpin residue (115 Å^2^ buried) entrapped by a phenolphosphonate pair. This type of lysine – **pclx_6_** interface (with cation−*π* interactions and salt bridges) occurred in two previous structures with cytochrome *c* and may be considered a supramolecular synthon (Figure S1).^[^ ^13^, ^17^ ^]^

Structure superposition of the RSL – **pclx_6_** and the RSL – **sclx_8_** binding sites reveals an approximate overlap of 4 or 3 phenolic units at the Lys25/Lys83 and Val13/Lys34 sites, respectively (Figure 3B). At the former site, **pclx_6_** shifts *circa* 1.5–2.0 Å with respect to **sclx_8_**, while at the latter there is a less pronounced shift of < 1.5 Å. Nonetheless, the main features of the protein – calixarene binding sites are conserved. For example, the interactions of **pclx_6_** or **sclx_8_** with Lys34 and Pro44 are essentially identical. Models providing insight into why **pclx_6_** binds as it does, and the likelihood of alternative binding modes are provided in the supplementary information (Figures S2 and S3). At each binding site, the displacement of **pclx_6_** with respect to the position of **sclx_8_** appears to allow for optimal masking of the protein surface and increased noncovalent interactions (Figures 3B; S3). Furthermore, these interface rearrangements facilitate crystal packing.

### 2.2 Crystal Packing Assemblies

**Figure 4:**
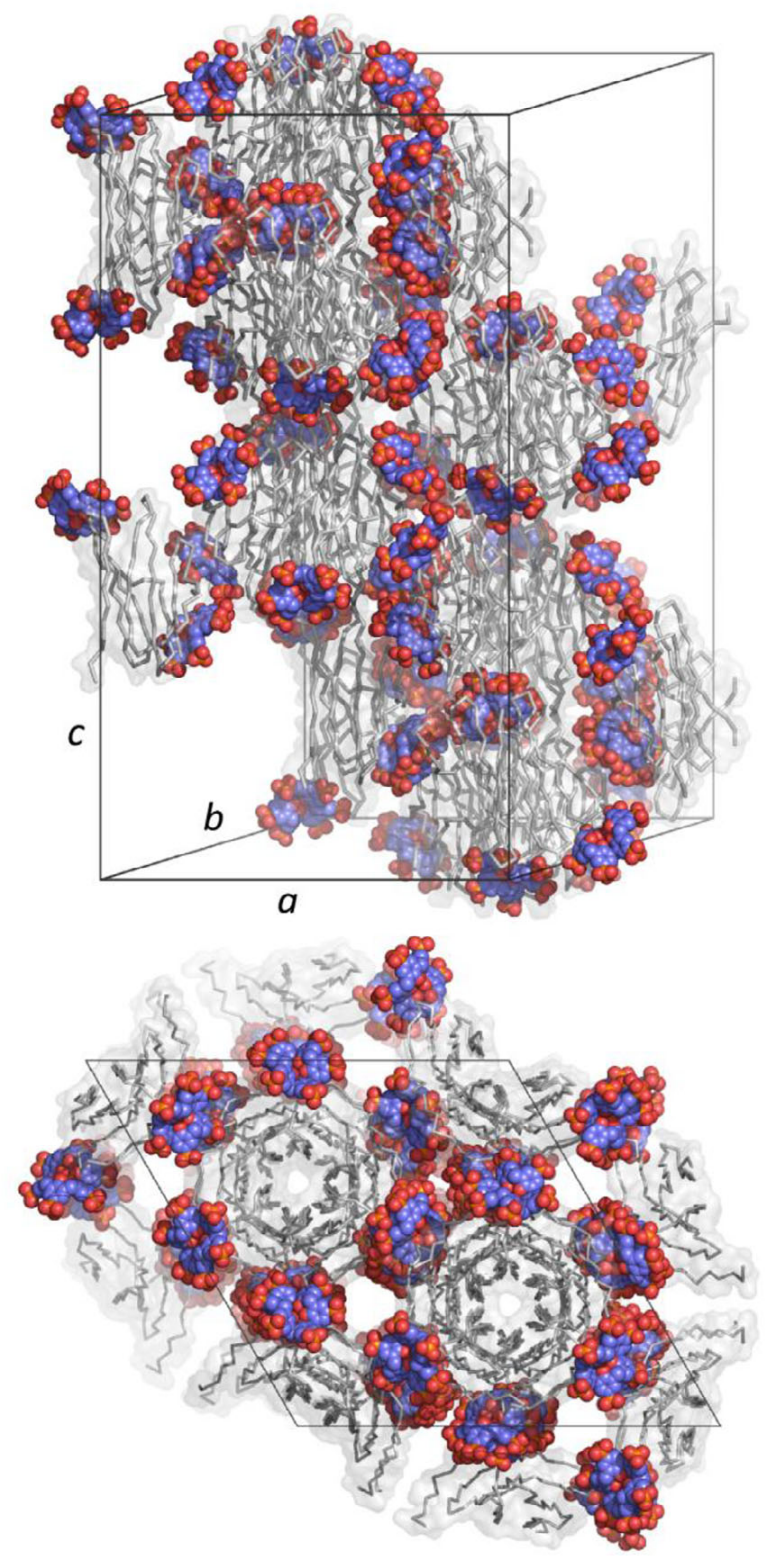
The unit cell of the RSL – **pclx_6_** cocrystal in space group *H*32.

While **pclx_6_** binding to the protein surface is predictable based on the known RSL – **sclx_8_** interfaces^[^ ^8^ ^]^ (Figures 3B; S3), the crystal packing is significantly altered. Structural differences in the interchangeable linkers result in different relative orientations of the protein nodes in the two structures, yielding different crystal symmetries. As a consequence of the *C*_2_‐symmetric double‐cone conformation, the **pclx_6_** – **pclx_6_** synthon can occur in two equivalent ways via 180° rotation (as observed in two cytochrome *c –* **pclx_6_** complexes).^[^ ^13^, ^17^ ^]^ Assuming that the **pclx_6_** – RSL binding sites are fixed, two relative orientations of the protein nodes are possible, depending on **pclx_6_** dimerization (Figure S4). Only one of these orientations occurs in the actual RSL – **pclx_6_** cocrystal structure. While the RSL – **sclx_8_** framework is mediated exclusively by the calixarene – calixarene synthon,^[^ ^8^ ^]^ the same is not possible with the **pclx_6_** dimer which would result in packing clashes as well as gaps in the crystal (Figure S5). Therefore, although crystal packing is predominantly mediated by **pclx_6_**, additional protein – protein contacts are required to assemble the calixarene‐linked protein nodes (Figure 4). In further contrast to the cubic RSL – **sclx_8_** form,^[^ ^8^ ^]^ the RSL – **pclx_6_** framework is nonporous (44% solvent content, Table 1 and Figure 4).

### 2.3 Synthon Sorting

Previously, we reported a ternary **sclx_8_** – cytochrome *c* – **pclx_6_** cocrystal mediated by reproducible protein – calixarene and calixarene – calixarene synthons.^[^ ^17^ ^]^ Separate **sclx_8_** and **pclx_6_** sites on the lysine‐rich cytochrome *c* surface allow for simultaneous complexation and formation of a ternary complex. In contrast, overlapping **sclx_8_**‐ and **pclx_6_**‐binding sites on RSL prevent ternary complexation utilizing the known RSL – calixarene interfaces (Figure S6).^[^ ^8^, ^18^ ^]^ Ternary mixtures of RSL, **sclx_8_**, and **pclx_6_** were subjected to cocrystallization trials to identify which of the competing calixarene – calixarene synthons (linkers) are preferred for framework fabrication. Ternary mixtures of 1 mm RSL, 10 mm **pclx_6_** and 10 mm **sclx_8_** yielded crystals in four conditions, (1) 20% PEG 3350, 0.2 m potassium nitrate, (2) 1.26 m ammonium sulfate, 0.1 m Tris‐HCl pH 8.5, 0.2 m lithium sulfate, (3) 0.8–1.2 m ammonium sulfate, 0.1 m sodium citrate pH 6 and (4) 0.8–1.2 m ammonium sulfate, 0.1 m sodium citrate pH 4. X‐ray diffraction data revealed pH‐driven self‐sorting,^[^ ^28^, ^29^ ^]^ with the RSL – **sclx_8_** cubes prevailing exclusively at pH 4. The RSL – **pclx_6_** cocrystals prevail without apparent competition in all other conditions (1–3), where the RSL – **sclx_8_** form is unstable (Figure S7). As depicted in Figure 1, the **sclx_8_** and **pclx_6_** dimers are stabilized by different intermolecular interactions. **pclx_6_** dimerization includes intermolecular hydrogen bonding in the phosphonate rim. This type of interaction is not possible with the fully deprotonated sulfonates of **sclx_8_**. While both forms can grow at pH ≤ 4, the RSL – **sclx_8_** cubes prevail in ternary trials, suggesting that **sclx_8_** is the better binder – consistent with its larger interface areas. However, **pclx_6_** apparently acted as a competitor, as evidenced by slower RSL – **sclx_8_** crystal growth with increased **pclx_6_** concentration (Figure S8).

### 2.4 Breaking the pclx_6_ – pclx_6_ Synthon

Polymorph synthesis and characterization is an economical means of generating diverse materials from a minimal set of building blocks. In the case of RSL – **sclx_8_** cocrystallization, there are at least four condition‐dependent arrangements of the protein and macrocycle.^[^ ^8^, ^18^, ^19^ ^]^ Three porous frameworks are obtained at pH 2–6 where RSL is cationic. These frameworks are directed exclusively by the calixarene, with no protein – protein contacts. The most porous structure is the cubic (*I*23) assembly directed by staggered **sclx_8_** dimers at pH ≤ 4. Changing the crystallization condition (e.g., pH) *breaks* the **sclx_8_** dimer and results in altered calixarene binding on the protein surface.

In contrast, the robust **pclx_6_** – **pclx_6_** synthon mediates a trigonal, nonporous assembly of RSL across a wide range of conditions (Table 1 and Figure 2). Since polymorph searching by screening crystallization conditions failed to produce different RSL – **pclx_6_** frameworks, we turned to protein engineering. As all three lysines of RSL participate in **pclx_6_** complexation (Figure 3), we hypothesized that an additional lysine was required to alter calixarene binding. Minimal affinity tags comprising 1–3 residues can function as macrocycle binding sites.^[^ ^16^, ^20^, ^21^, ^30^ ^]^ For example, Urbach and coworkers demonstrated that cucurbit[8]uril binds a series of Met‐terminated tripeptides with high affinity, including Met‐Lys‐Ala (*K*_d_ ≈3 µm).^[^ ^30^ ^]^ Later, we discovered that cucurbit[6]uril binds the highly accessible N‐terminal Met‐Lys motif in the protein SAMP2.^[^ ^16^ ^]^ Transfer of this macrocycle binding site to RSL, in the variant MK‐RSL (p*I* ≈7.8), facilitated complexation with cucurbit[6]uril or **sclx_4_**.^[^ ^16^, ^21^ ^]^ The three N‐termini, in close proximity and protruding from the narrow end of the MK‐RSL toroid, are usually disordered in crystal structures.

**Figure 5:**
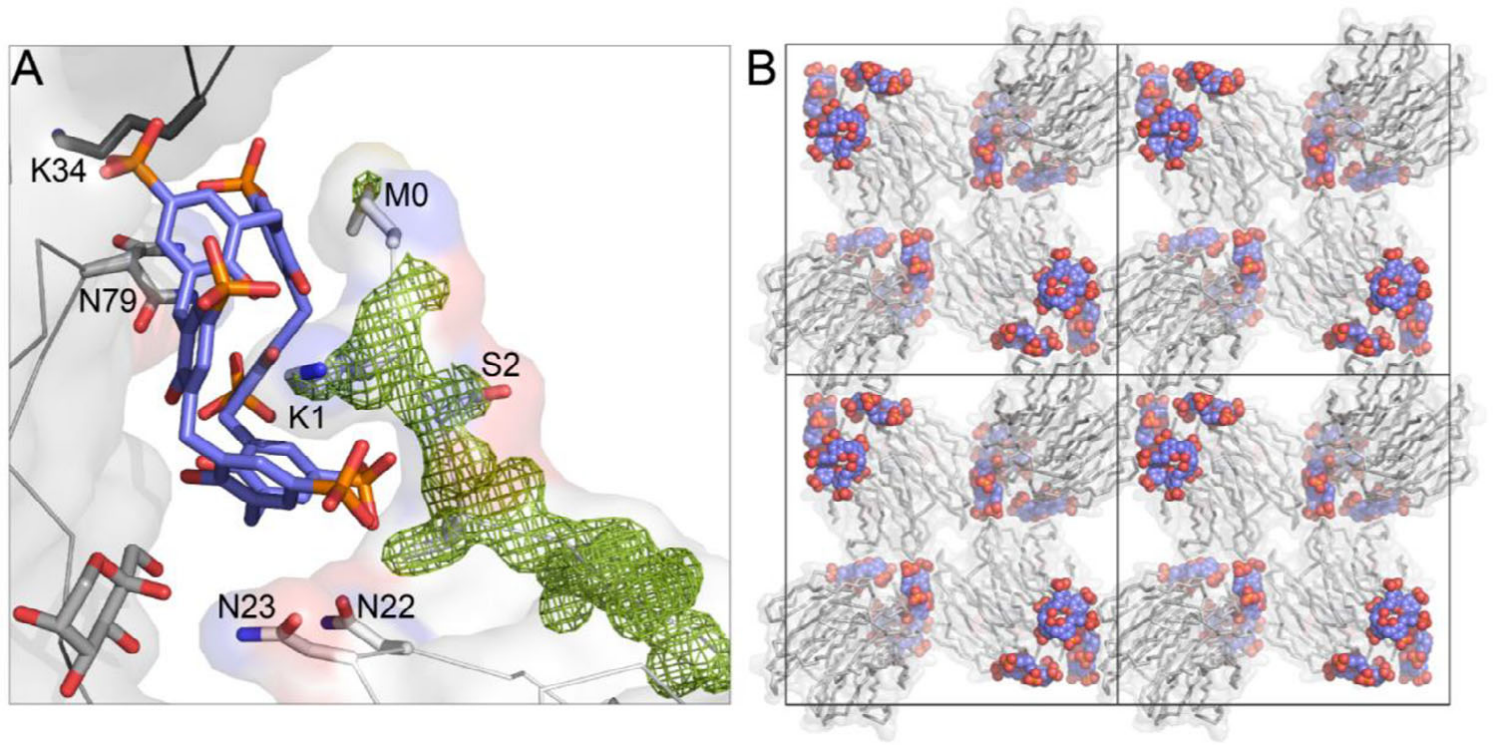
The MK‐RSL – **pclx_6_** cocrystal structure. **A) pclx_6_** in the double cone conformation complexes Lys1, as evidenced in the 2Fo‐Fc electron density maps (contoured at 1.0 σ, green mesh). **B)** Crystal packing in the cubic framework (space group *I*23).

MK‐RSL – **pclx_6_** cocrystals with truncated octahedral or cubic morphology (Figure S9) grew reproducibly within 1–2 days in two conditions, 1) 1.26 m ammonium sulfate, 0.1 m Tris‐HCl, pH 8.5, 0.2 m lithium sulfate and 2) 1.0 m di‐ammonium hydrogen phosphate, 0.1 m sodium acetate, pH 4.5. The crystals diffracted to 1.2 Å resolution and were solved in the cubic space group *I*23 (Table S2; Figure 5). As predicted, the N‐terminal Met‐Lys motif functioned as a **pclx_6_** binding site. In contrast to previous protein – **pclx_6_** structures,^[^ ^13^, ^17^ ^]^ the dimeric **pclx_6_** – **pclx_6_** synthon does not occur. Instead, a monomeric **pclx_6_** binds and *freezes* each Met‐Lys motif. The calixarene adopts the 1,2,3‐alternate double cone conformation, affording residue encapsulation in two cones formed by three phenolphosphonates (Figure 5A). Lys1 is entrapped in one cone, with the C**^ε^** forming cation–*π* interactions with two phenolic rings and the N^ζ^ ammonium salt‐bridging two phosphonates. This binding mode allows for increased encapsulation of the lysine residue (≈155 Å^2^ buried) compared to the shallow binding pockets of the **pclx_6_** dimer (≈115 Å^2^ of Lys34 buried in RSL – **pclx_6_**). Notably, the lysine – **pclx_6_** interface duplicates a previously described lysine – **sclx_6_** synthon (Figure S10).

The other half of **pclx_6_** buries ≈100 Å^2^ of Asn79 on another RSL trimer (Figure 5A). The Asn79 side chain adopts alternate conformations, suggesting dynamic binding and lower importance in complex formation compared to Lys1. Notably, the **pclx_6_** binding sites in the *H*32 RSL structure are lost. Instead, the monomeric **pclx_6_** directs a cubic *I*23 framework with 53% solvent content (Figure 5B). In addition to the protein – calixarene sites, a single protein – protein interface (290 Å^2^) contributes to the crystal packing. An isomorphous structure was obtained with PK‐RSL containing a Pro‐Lys N‐terminal extension (Figures S11 and S12 and Tables S2 and S3). Residue0 (Met or Pro) is partially disordered in each case, suggesting that Lys1 is crucial in directing **pclx_6_** binding. Nonetheless, the N‐terminal ammonium forms a (water‐mediated) salt bridge to a phosphonate of a neighboring **pclx_6_** bound on the same protein trimer, contributing to the assembly. Adding the MK or PK tag at the disordered N‐terminus does not result in any notable changes in the protein backbone or at the original RSL – **pclx_6_** binding sites (Figures S13 and S14). As such, the observed differences in the crystal structure result solely from preferential **pclx_6_** binding at the N‐terminal tag.

**Figure 6:**
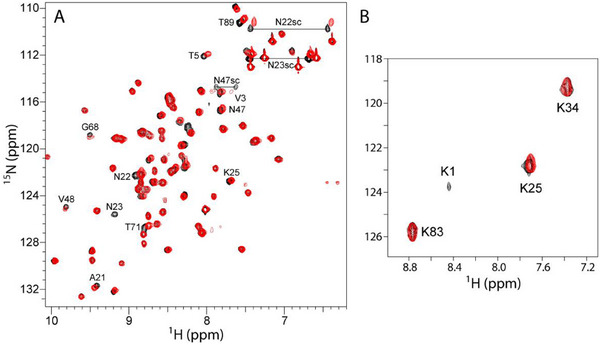
**^1^** H−^15^N HSQC spectra (at 30 °C) of 0.1 mm A) ^15^N‐labelled MK‐RSL or B) ^15^N‐lysine‐labeled MK‐RSL in the absence (black contours) or presence (red) of 0.3 mm **pclx_6_**, in 20 mm potassium phosphate, 50 mm NaCl, 5 mm D‐fructose, 10% D_2_O, pH 6.0.

The dicationic Met‐Lys N‐terminus alters **pclx_6_** binding in the solid and solution states. **^1^** H−^15^N HSQC NMR spectroscopy was used to monitor **pclx_6_** binding in solution. RSL and **pclx_6_** have negligible interactions in solution at pH 6 (Figure S15). In the same conditions, the **^1^** H−^15^N HSQC spectrum of MK‐RSL is altered upon the addition of 0.3 mm **pclx_6_** (Figure 6). Residues near the N‐terminal binding site, Val3, and Thr5, are upfield shifted. Ala21, Asn22, Asn23, Gly68, and Thr71, are also shifted or broadened beyond detection (Asn23). In the crystal structure, Asn22, Asn23 (Figure 5A), and Gly68 have van der Waals contacts with the **pclx_6_** periphery. There were no further chemical shift perturbations upon increasing **pclx_6_** concentration to 0.6 mm, suggesting that the MK‐RSL – **pclx_6_** binding sites were saturated at 3 equivalents of the calixarene. A lysine‐labeled sample was used to analyze further the **pclx_6_** interactions. Complexation at the accessible Lys1 is suggested by resonance broadening, with negligible interactions at the other three lysines (Figure 6). Enhanced solution state interactions are consistent with increased lysine masking in the crystal structure (Figure 5A). Thus, the NMR experiments confirm that both the location and mode of **pclx_6_** binding are altered on MK‐RSL compared to wild‐type RSL. Minimal protein engineering results in selective Lys1 binding, *breaking* the robust **pclx_6_** dimer and yielding a new framework.

## 3 Conclusions

Water soluble calixarenes in combination with proteins yield crystalline frameworks, serving as a basis for biomaterials. Progress in crystal engineering can be facilitated by identifying and applying supramolecular synthons.^[^ ^8^, ^13^, ^15^, ^17^, ^18^, ^19^, ^22^ ^]^ Motivated by modular crystal synthesis,^[^ ^1^, ^2^, ^22^ ^]^ we demonstrated the possibility of interchangeable modules in protein – calixarene frameworks by swapping supramolecular synthons. Such modularity is desirable to generate frameworks with predictable structures and tunable functions. For example, despite similar protein recognition properties, substituting the **sclx_8_** dimer for the **pclx_6_** dimer yielded a framework that is stable across a wider range of conditions (Table 1) due to altered calixarene self‐assembly. Notably, the RSL – **sclx_8_** and RSL – **pclx_6_** frameworks demonstrated condition‐dependent self‐sorting, which is potentially useful in complex systems.

Over the past decade, modular protein crystal engineering has advanced significantly. Tezcan and coworkers generated a series of ferritin MOFs by swapping interchangeable components (metals or organic linkers).^[^ ^3^, ^6^ ^]^ Mirkin and coworkers tuned Concanavalin A‐based frameworks via mannose‐DNA linkers of varied lengths or sequences.^[^ ^9^ ^]^ More recently, Baker and coworkers engineered protein crystal porosity by altering the length of *de novo* designed protein components.^[^ ^10^ ^]^ However, each of these systems required either extensive protein engineering or specific metal/ligand binding sites. In contrast, calixarenes provide a simple yet versatile alternative, compatible with various protein targets.^[^ ^8^, ^13^, ^15^, ^17^, ^18^, ^19^ ^]^ By expanding the library of protein – calixarene frameworks, and identifying and applying synthons, we can develop a protein crystal engineering toolkit affording the development of new types of hierarchical biomaterials.

Polymorphs arise when the binding mode and location of the flexible calixarene on a protein surface varies depending on the crystallization conditions. Regardless of extensive screening, a single RSL– **pclx_6_** cocrystal was obtained, mediated by the robust calixarene dimer. In contrast, four RSL – **sclx_8_** polymorphs have been identified, three of which are pH‐sensitive.^[^ ^8^, ^18^ ^]^ To produce a new framework, molecular interactions were altered via simple protein engineering. Including an accessible lysine (MK‐RSL) was sufficient to engage **pclx_6_** in a new way, *breaking* the durable calixarene dimer. A porous framework was generated with minimal alterations of the protein component. Considering this result, it is feasible that the N‐terminal Met‐Lys binding tag may be applied to design protein – macrocycle frameworks for other proteins of interest. Minimal protein tags (1–3 residues) at terminal locations are of particular interest.^[^ ^20^, ^21^ ^]^ Such engineering may afford the tailored assembly of proteins, such as enzymes, without disrupting the structure/function relationship. The application of macrocycle binding tags may also facilitate the co‐assembly of different protein types, serving as a tool for fabricating functional materials.

## Author Contributions

N.M.M. contributed to the project concept, sample preparation, data collection and analysis, manuscript writing, and figure preparation; C.L.R. was responsible for sample preparation and manuscript review; P.B.C. contributed to the project concept, data collection and analysis, manuscript writing and figure preparation.

## Conflict of Interests

The authors declare no conflict of interest.

## Supporting information



Supporting Information

## Data Availability

Crystal structure data are available from the Protein Data Bank (PDB 9hbd, 9hbe, 9hbf and 9hbg), additional data are available in the ESI or from the corresponding author.
