# Supplementary material for: Making and Breaking Supramolecular Synthons for Modular Protein Frameworks
Source: Chemistry. 2025 Apr 16;31(28):e202500732. doi: 10.1002/chem.202500732 (PMC12089892; doi:10.1002/chem.202500732)
Supplement: Supplementary file 1 — Supporting Information [file CHEM-31-e202500732-s001.docx]

*Supplementary* *Information*

**Making and Breaking Supramolecular Synthons for Modular Protein Frameworks**

Niamh M. Mockler,^[a]^ Colin L. Raston,^[b]^ and Peter B. Crowley*^,[a]^

^[a]^ School of Biological and Chemical Sciences, University of Galway, University Road, Galway

H91 TK33, Ireland.

^[b]^ Flinders Institute for Nanoscale Science and Technology, College of Science and Engineering, Flinders University, Bedford Park SA 5042, Adelaide, Australia

*Correspondence to: peter.crowley@universityofgalway.ie +353 91 49 24 80

**Contents**

Experimental S2

Table S1 RSL – **pclx_6_** X-ray data collection, processing and refinement statistics S5

Figure S1 Lysine – **pclx_6_** dimer, supramolecular synthon S6

Figure S2 Superposition of pleated **sclx_8_** and double-cone **pclx_6_** S7

Figure S3 **pclx_6_** binding models (true and hypothetical) S8

Figure S4 RSL – **pclx_6_** assembly models (true and hypothetical) S9

Figure S5 RSL – **pclx_6_** packing models (hypothetical) S10

Figure S6 RSL – **pclx_6_** and RSL – **sclx_8_** cocrystal forms S11

Figure S7 RSL – **pclx_6_** crystals in ternary crystallization drops S12

Figure S8 RSL – **sclx_8_** crystals in ternary crystallization drops S13

Figure S9 MK-RSL – **pclx_6_** cocrystals S14

Table S2 XK-RSL – **pclx_6_** X-ray data collection, processing and refinement statistics S15

Figure S10 Lysine – calix[6]arene, supramolecular synthon S16

Figure S11 ESI+ mass spectrum of PK-RSL S17

Table S3 Predicted and measured masses of PK-RSL S17

Figure S12 PK-RSL – **pclx_6_** cocrystal structure S18

Figure S13 Structural superimposition of RSL and MK-RSL trimers S19

Figure S14 Structural superimposition of RSL and MK-RSL at RSL – **pclx_6_** binding sites S20

Figure S15 **^1^**H−^15^N HSQC spectra of RSL in the absence or presence of **pclx_6_** S21

References S22

**Experimental**

**Materials.** Stock solutions of the *p-*phosphonato-calixarenes,^[1,2]^ or *p*-sulfonato-calixarenes (Tokyo Chemical Industry) were prepared in milliQ water at pH 7–8. The pET25rsl vectors encoding RSL and MK-RSL were reported previously^[3,4]^ and the vector encoding PK-RSL was produced by Genscript. Unlabeled, ^15^N-labeled and ^15^N-lysine-labeled proteins were expressed in *E. coli* BL21 transformed with the relevant plasmid.^[3]^ RSL and variants were purified by mannose affinity chromatography and exchanged into water (RSL) or 20 mM potassium phosphate, 50 mM NaCl, pH 6.0 (MK-RSL and PK-RSL).^[3,4]^ Protein concentration was determined spectrophotometrically with ε_280_ = 44.46 mM^−1^ cm^−1^ for the monomer. Mass analysis was performed with an Agilent 6530 Accurate-Mass Q-TOF LC/MS with 40 µM PK-RSL in water. *Saccharomyces cerevisiae* cytochrome *c* was produced and purified as described.^[5]^

**Cocrystallization Trials.** Crystallization trials were prepared at 20 °C using D-fructose-bound RSL, MK-RSL or PK-RSL. Binary mixtures comprised 1 mM protein and 0.5-32 mM calixarene. Sitting drop vapour diffusion experiments were prepared in MRC plates using a commercial screen (JCSG++ HTS, Jena Bioscience) and dispensed with an Oryx8 robot (Douglas Instruments). Cocrystals were also produced via hanging drop vapor diffusion experiments in 24 well Greiner plates. RSL – **pclx_6_** mixtures were tested in homemade conditions comprising precipitant (PEG 3350 or ammonium sulfate), 0.1 M buffer (sodium citrate pH 4-6 or Tris-HCl pH 8.5) plus additive (0-0.2 M lithium sulfate). Conditions comprising only sodium citrate (0.4-1.2 M at pH 5.0 or 6.0) or 20 mM sodium acetate pH 4.0 plus 50-100 mM NaCl were also tested. All conditions that previously yielded RSL – **sclx_8_** polymorphs were screened.^[3,6]^ RSL was tested with *p*-phosphonato-calix[8]arene (**pclx_8_**) or *p*-sulfonato-calix[6]arene (**sclx_6_**) in similar conditions. Ternary mixtures comprising 1 mM RSL, 2-10 mM **pclx_6_** and 2-10 mM **sclx_8_** were tested using the commercial screen or homemade screens comprising 0.6-1.2 M ammonium sulfate and 0.1 M citrate at pH 4.0, 5.0 or 6.0. Cytochrome *c* – **sclx_8_** mixtures comprising 1 mM protein, 0.5-20 mM **sclx_8_** and 0-5 mM ascorbate were trialed via hanging drop vapour diffusion. The conditions comprised precipitant (ammonium sulfate), 0.1 M buffer (sodium citrate pH 4 or 5) plus additive (0-0.2 M NaCl). Conditions comprising only sodium citrate or sodium malonate (0.4-1.4 M at pH 5 or 6) were also tested. Trials were prepared in the presence or absence of seeds of cytochrome *c* – **sclx_8_** cocrystals^[7]^ or Na–**sclx_8_** rods.^[8]^

**X-ray Data Collection, Processing, and Model Building.** Crystals were cryo-protected in the crystallization solution supplemented with 25% glycerol and cryo-cooled in liquid nitrogen. Diffraction data were collected at beamline PROXIMA-2A, SOLEIL synchrotron (Saint-Aubin, France) with an Eiger X 9M detector. Data were processed using the autoPROC pipeline,^[9]^ with integration in XDS^[10]^ followed by scaling and merging in AIMLESS^[11]^ and POINTLESS^[12]^ in CCP4. The structures were solved by molecular replacement in PHASER^[13]^ using the RSL monomer (PDB 2bt9) as the search model. Coordinates and restraints for **pclx_6_** (7AZ) and D-fructose (BDF) or glycerol (GOL) were added to the models in COOT.^[14]^ Model building in COOT and refinement in PHENIX^[15]^ were continued iteratively until the electron density and R_free_ could be improved no further. Structures were validated in MolProbity^[16]^ and deposited in the Protein Data Bank with accession codes 9hbd (RSL – **pclx_6_** at pH 4), 9hbe (RSL – **pclx_6_** at pH 8.5), 9hbf (MK-RSL – **pclx_6_**) and 9hbg (PK-RSL – **pclx_6_**). Protein − ligand interface areas were measured in PDBe PISA^[17]^ and crystal pore diameters were calculated in MAP_CHANNELS.^[18]^ X-ray data collection, processing and refinement statistics are listed in Tables S1 and S2.

**NMR Characterization.** ^1^H–^15^N HSQC-monitored titrations were performed at 30 °C using a 600 MHz Varian spectrometer equipped with a HCN cold probe, as described.^[3,4]^ NMR samples comprised 0.1 mM ^15^N-labeled or ^15^N-lysine-labeled protein in 20 mM potassium phosphate, 50 mM NaCl, 5 mM D-fructose, 10% D_2_O, pH 6.0. Samples were titrated with 1.5 µL aliquots of 100 mM **pclx_6_**. The pH of the sample was adjusted to pH 6.1 after each addition of ligand.

**Binding / packing model building (Figures S2**–**S5).** Each face of a pleated **sclx_8_** presents four shallow cavities (*binding pockets*) formed by pairs of phenolsulfonates.^[3]^ One face mediates calixarene dimerization and the other face has four pockets available to bind residues on the protein surface (Figure S2). In contrast, the two faces of a double-cone **pclx_6_** differ. The face that mediates **pclx_6_** dimerization presents two cone-like cavities, each formed by three phenolphosphonates. The other face has two shallow cavities formed by pairs of phenolphosphonates, analogous to the **sclx_8_** binding pockets (Figure S2). A pleated **sclx_8_** and double cone **pclx_6_** can be superposed such that one binding pocket overlaps (Figure S2)**.** Models based on superposing the calixarenes assessed the predictability of **pclx_6_** binding at the Lys25/Lys83 and Val13/Lys34 sites on RSL (Figure S3). Models were built in COOT with two assumptions: (1) **pclx_6_** adopts the double-cone conformation necessary for dimerization and (2) **pclx_6_** binding maintains features of **sclx_8_** binding. Four potential **pclx_6_** binding modes were considered at each **sclx_8_** binding site by sequentially superposing binding pockets, *i.e.* a pair of phenolphosphonates was aligned with each pair of phenolsulfonates (Figure S3). Each model was evaluated by analyzing the noncovalent protein – calixarene interactions or absences, as well as clashes that could not be alleviated by a simple side chain rotamer, maintaining or impeding complexation respectively (Figure S3). Protein – calixarene assembly models (crystal packing) were built in COOT with two assumptions: (1) The protein – calixarene interfaces are fixed and (2) the calixarene – calixarene synthons (dimers) are formed (Figures S4 and S5).

**Table S1**. X-ray data collection, processing and refinement statistics for the RSL – **pclx_6_** crystal form.

| **Crystallization** | | | | | |
| --- | --- | --- | --- | --- | --- |
| **[RSL] (mM)** | 1 | 1 | 1 | 1 | 1 |
| **[pclx_6_] (mM)** | 32 | 32 | 10 | 10 | 10 |
| **[sclx_8_] (mM)** | - | - | - | 10 | 10 |
| **Precipitant** | 18%  PEG 3350 | 0.4 M ammonium sulfate | 0.6 M  sodium  citrate | 0.8 M ammonium sulfate | 1.26 M ammonium sulfate |
| **Buffer (0.1 M)** | Na-citrate  pH 4.0 | Na-citrate  pH 4.0 | pH 5.0 | Na-citrate  pH 6.0 | Tris-HCl  pH 8.5 |
| **Additive (0.2 M)** | - | - | - | - | Li_2_SO_4_ |
| **Data Collection***^a^* | | | | | |
| **Light Source** | SOLEIL, PROXIMA-2A | | | | |
| **Wavelength (Å)** | 0.98011 | | | | |
| **Space group** | *H*32 | | | | |
| **Cell constants (Å)** | 82.991  82.991 157.427 | 82.963  82.963 158.100 | 83.678  83.678 157.677 | 83.620  83.620  158.056 | 83.149  83.149  157.813 |
| **Resolution (Å)** | 52.48–1.09 (1.11–1.09) | 52.70–1.16 (1.18–1.16) | 52.56–1.12 (1.14–1.12) | 52.69–1.15 (1.17–1.15) | 53.19–1.19 (1.21–1.19) |
| **# reflections** | 1354877 (9206) | 1114522 (19062) | 1350523 (17131) | 1326358 (23181) | 1159073 (29591) |
| **# unique reflections** | 82159  (2190) | 72365  (3448) | 79182  (3156) | 74939  (3158) | 67645  (3253) |
| **Multiplicity** | 16.5 (4.2) | 15.4 (5.5) | 17.1 (5.4) | 17.7 (7.3) | 17.1 (9.1) |
| **I/σ (I)** | 16.7 (0.9) | 31.9 (2.2) | 11.2 (0.5) | 9.1 (0.9) | 8.5 (0.7) |
| **Completeness (%)** | 93.9 (50.5) | 99.6 (95.7) | 97.6 (78.8) | 98.4 (84.3) | 99.9 (97.7) |
| ***R*_meas_*^b^* (%)** | 7.6 (181.3) | 4.0 (66.3) | 10.0 (299.4) | 15.6 (266.5) | 14.5 (352.4) |
| ***R*_pim_*^c^* (%)** | 1.7 (86.4) | 1.0 (26.9) | 2.3 (122.2) | 3.5 (92.3) | 3.4 (108.8) |
| **CC_1/2_** | 0.999 (0.452) | 1.000 (0.849) | 0.999 (0.407) | 0.995 (0.485) | 0.999 (0.532) |
| **Solvent content (%)** | 44 | | | | |
| **Refinement** | | | | | |
| ***R*_work_** | 0.184 | 0.178 | 0.178 | 0.176 | 0.177 |
| ***R*_free_** | 0.199 | 0.196 | 0.188 | 0.197 | 0.194 |
| **rmsd bonds (Å)** | 0.004 | 0.004 | 0.004 | 0.004 | 0.004 |
| **rmsd angles (°)** | 0.860 | 0.839 | 0.809 | 0.829 | 0.817 |
| **# molecules in asymmetric unit** | | | | | |
| **Protein chains** | 2 | | | | |
| **pclx_6_** | 4 | | | | |
| **water** | 324 | 317 | 328 | 343 | 272 |
| **Ave. B-factor (Å^2^)** | 19.21 | 20.34 | 19.24 | 13.25 | 14.97 |
| **Clashscore** | 0.64 | 0 | 0.65 | 0.32 | 0.65 |
| **Ramachandran analysis,*^d^* % residues in** | | | | | |
| **favoured regions** | 99.43 | 98.30 | 98.86 | 99.43 | 98.86 |
| **allowed regions** | 0.57 | 1.70 | 1.14 | 0.57 | 1.14 |
| **PDB code** | 9hbd | - | - | - | 9hbe |

*^a^*Values in parentheses correspond to the highest resolution shell; *^b^R*_meas_ = ∑*_hkl_* √(*n/n-1*)∑_i_ |*I_i_*(*hkl*) *-* 〈*I*(*hkl*)〉|/∑*_hkl_* ∑*_i_I_i_*(*hkl*); *^c^R*_pim_ = ∑*_hkl_* √(1/n-1)∑^n^ *_i=1_* |*Ii*(*hkl*) - 〈*I*(*hkl*)〉|/∑*_hkl_* ∑*_i_I_i_*(*hkl*); *^d^*Calculated in MolProbity


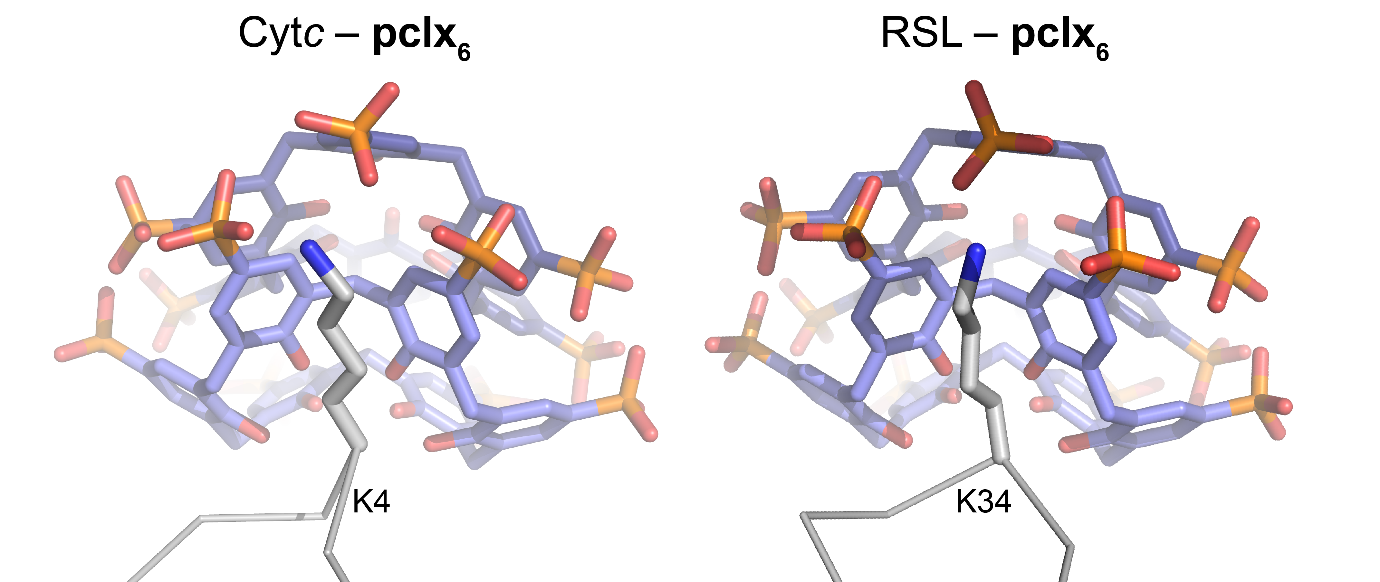


**Figure S1.** The lysine – **pclx_6_** synthon in cocrystal structures with cytochrome *c* (PDB 5lyc)^[19]^ or RSL (PDB 9hbd). A shallow phenolphosphonate *binding* *pocket* traps one linchpin lysine residue.


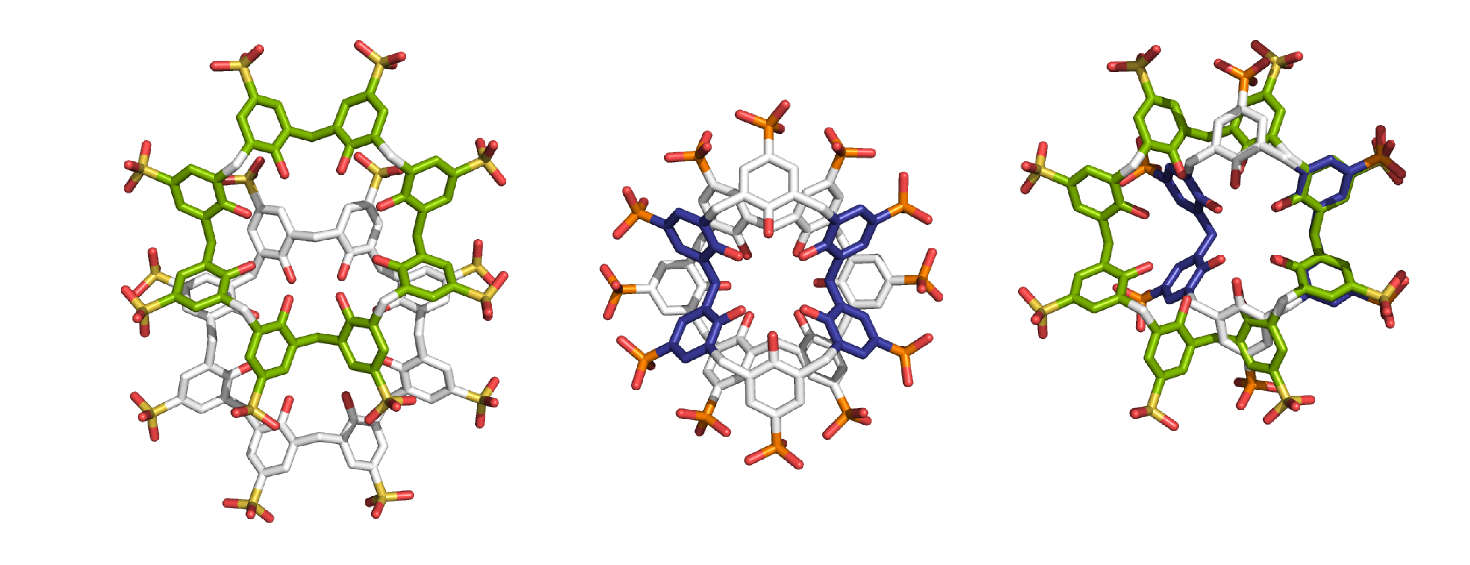


**Figure S2.** **Calixarene superposition.** In the **sclx_8_** dimer, each calixarene has four pairs of phenolic units (binding pockets, green) available to encapsulate protein residues. In the **pclx_6_** dimer, each calixarene has two such binding pockets (blue). A double-cone **pclx_6_** can be superposed on a pleated **sclx_8_** such that one binding pocket aligns precisely, while the remaining subunits have varying degrees of mismatch. Models A-H in Figure S3 were built using this alignment strategy.

**
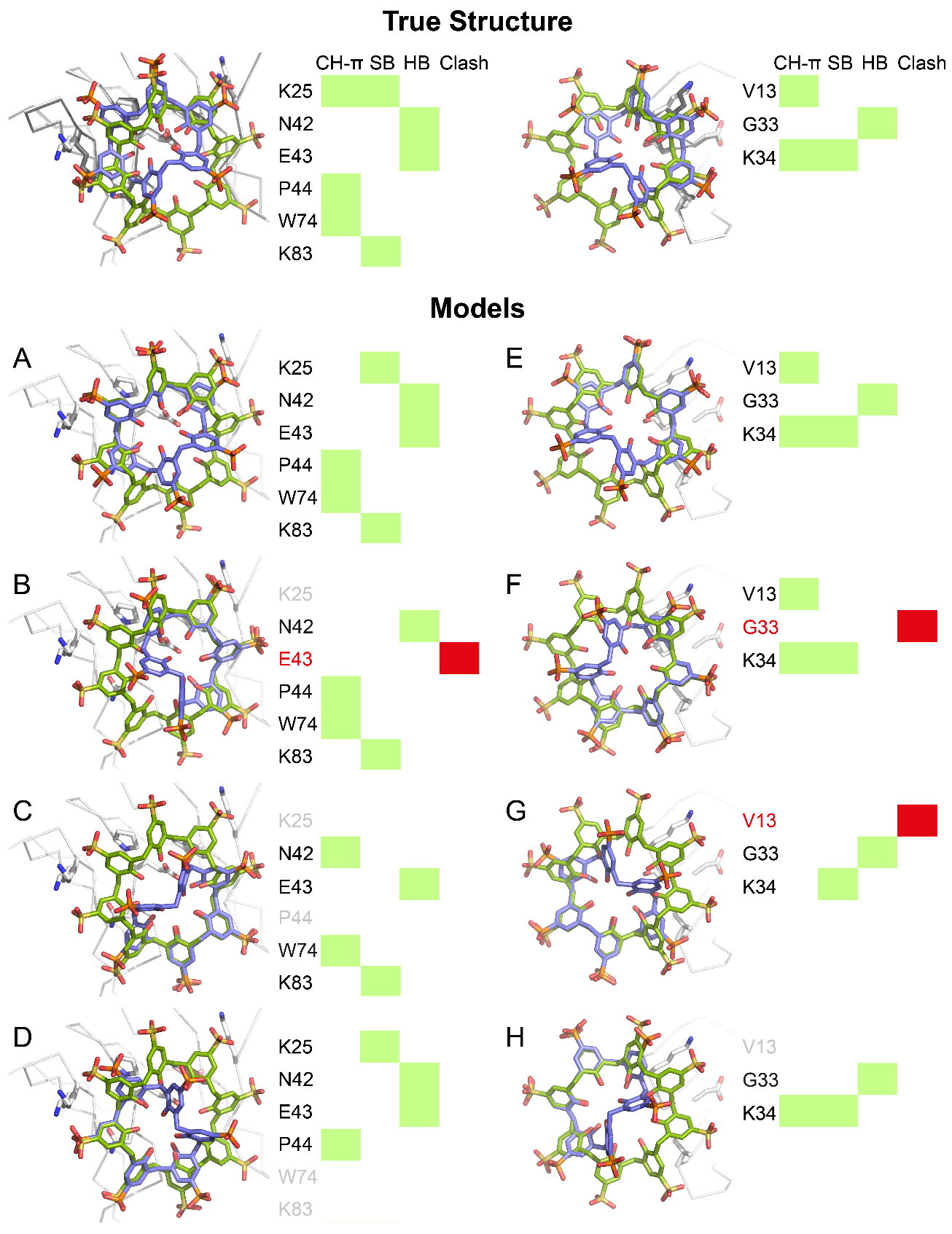
**

**Figure S3.** **Binding models**. Using the two RSL – **sclx_8_** interfaces as a scaffold, hypothetical binding models **A**–**H** were built by superposing **pclx_6_** onto **sclx_8_**. Potential noncovalent interactions (CH−*π*, salt-bridges, and hydrogen bonds) are denoted green. Missing interactions and steric clashes are denoted grey and red, respectively. Models **A** and **E** are satisfactory approximations of each binding site. The true interfaces vary from the models due to the imperfect overlap of **pclx_6_** and **sclx_8_** binding pockets.


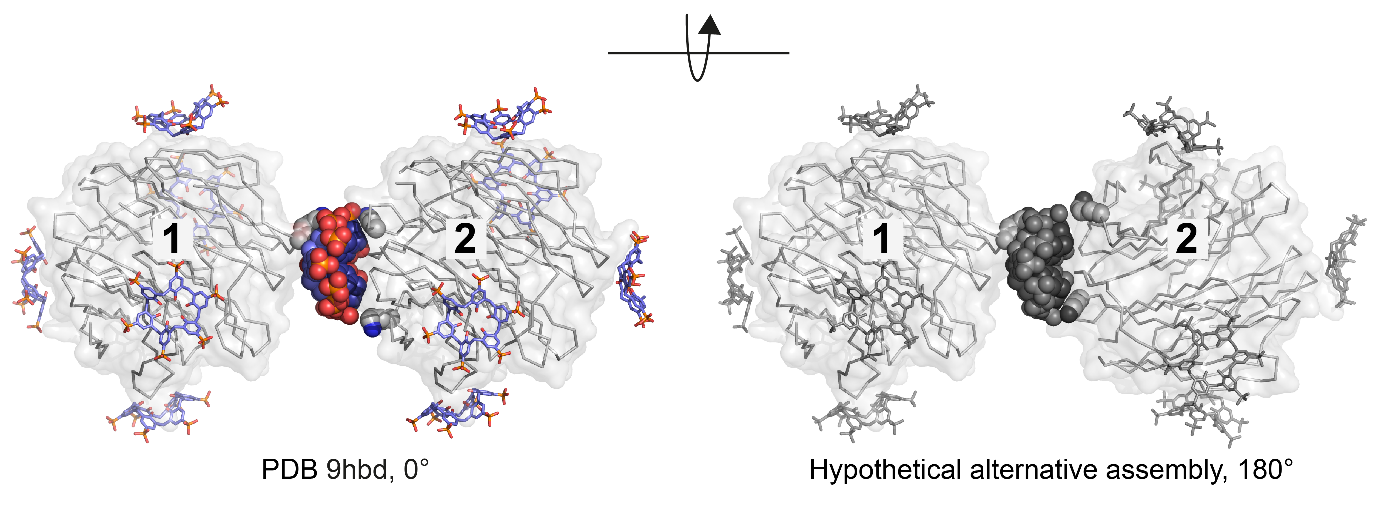


**Figure S4.** True (PDB 9hbd, coloured) and hypothetical (greyscale) alternate RSL – **pclx_6_** assemblies. The RSL – **pclx_6_** units (1 and 2) can combine in two possible ways differing by 180° rotation. Here, unit **1** is ‘fixed’, while unit **2** rotates by 180° around the calixarene dimer.


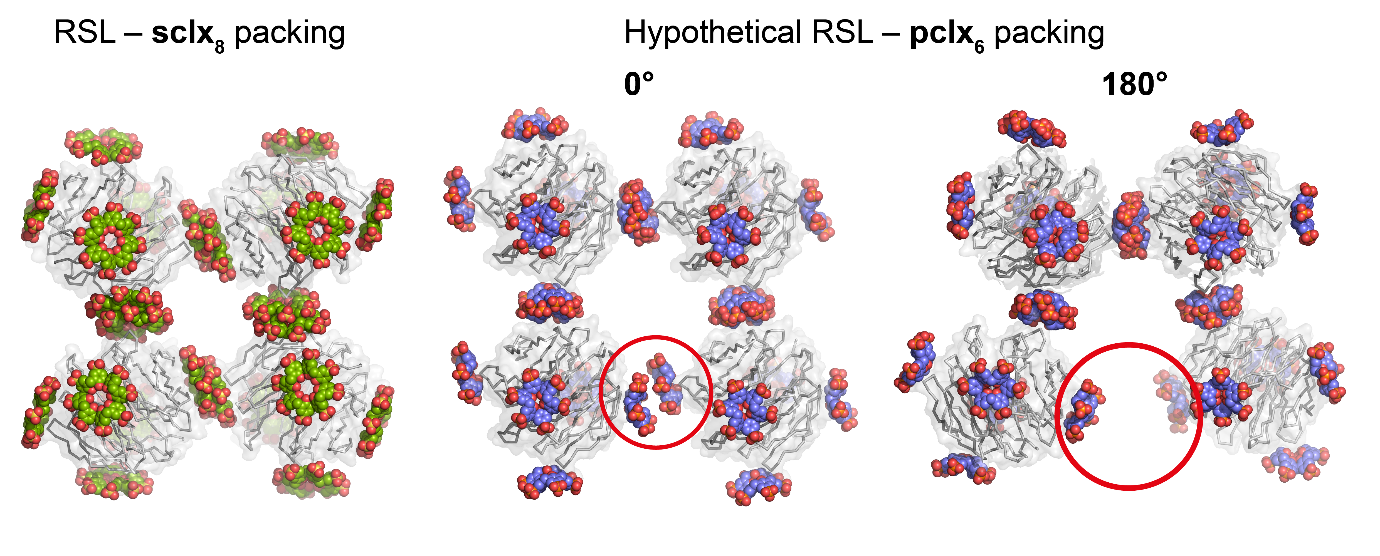


**Figure S5.** The cubic RSL – **sclx_8_** framework (*I*23 form) is mediated exclusively by the **sclx_8_** dimer, with no protein – protein contacts.^[3]^ Here, half of the unit cell is shown. This assembly is not possible with the disc-shaped **pclx_6_** dimer, as gaps (circled) and packing clashes (not shown) would occur. The hypothetical packing models were generated using the two possible RSL – **pclx_6_** dimerization modes depicted in Figure S4.


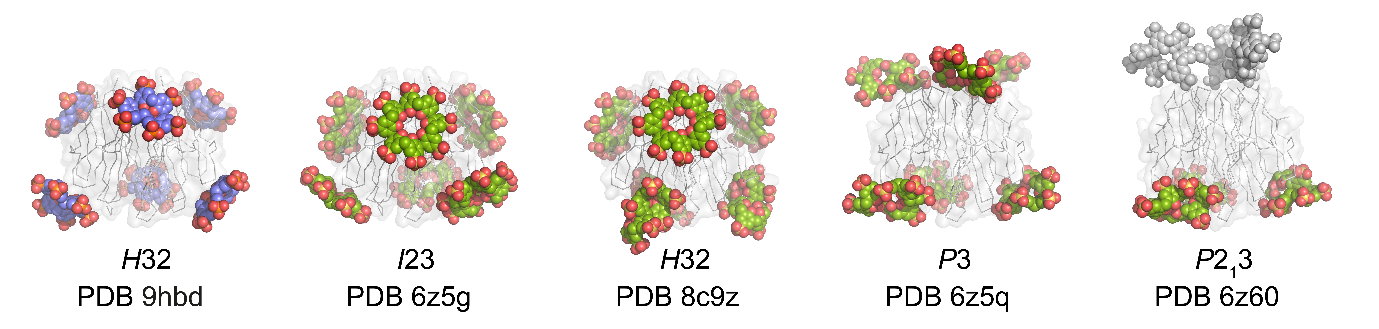


**Figure S6.** Calixarene binding at similar sites in the RSL – **pclx_6_** (mauve) and RSL – **sclx_8_** (green) cocrystal forms.^[3,6]^ Symmetry mates are coloured grey.


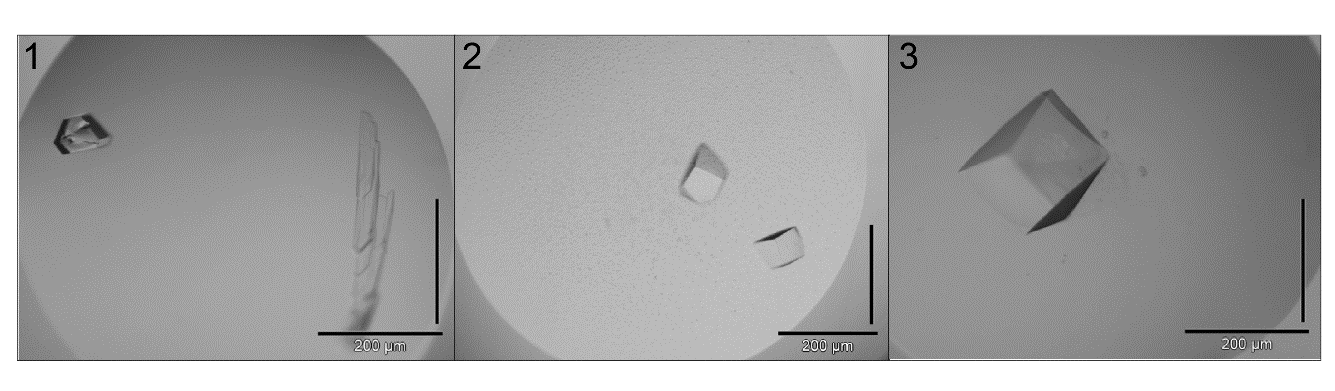


**Figure S7. Synthon sorting.** Ternary mixtures (1 mM RSL, 10 mM **pclx_6_** and 10 mM **sclx_8_**) yielded RSL – **pclx_6_** crystals in three conditions, (1) 20% PEG 3350, 0.2 M potassium nitrate, (2) 1.26 M ammonium sulfate, 0.1 M Tris-HCl pH 8.5, 0.2 M lithium sulfate and (3) 0.8–1.2 M ammonium sulfate, 0.1 M sodium citrate pH 6. Note, the thin plates in condition 1 were not tested as their morphology did not match either RSL – **sclx_8_** or RSL – **pclx_6_**.


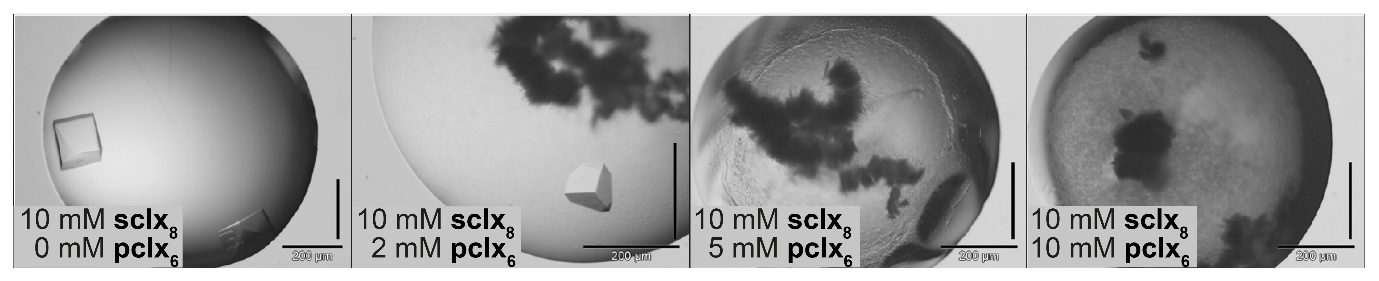


**Figure S8. Synthon sorting.** Ternary crystallization drops comprising 1 mM RSL, 10 mM **sclx_8_** and 0–10 mM **pclx_6_** in ~1 M Ammonium sulfate, 0.1 M sodium citrate pH 4. Pictures taken 6 days after drop set up. The dark precipitate is characteristic of this condition and leads to crystals. Cubic RSL – **sclx_8_** cocrystals are present in the two leftmost drops. Cubic RSL – **sclx_8_** cocrystals were found in the two rightmost drops on the day of harvesting, however, no picture was taken.

**
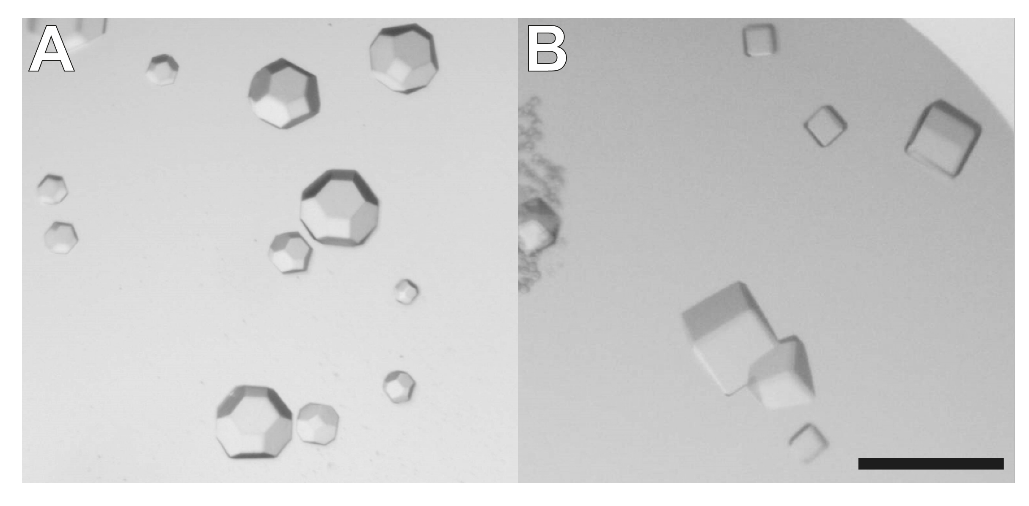
**

**Figure S9.** MK-RSL – **pclx_6_** cocrystals grown in **(A)** 1.26 M ammonium sulfate, 0.1 M Tris-HCl, pH 8.5, 0.2 M lithium sulfate and **(B)** 1 M di-ammonium hydrogen phosphate, 0.1 M sodium acetate, pH 4.5. Scale bar is 200 µm, for both images.

**Table S2**. X-ray data collection, processing and refinement statistics for XK-RSL – **pclx_6_** cocrystals.

| **Crystallization** | | |
| --- | --- | --- |
| **protein (1 mM)** | MK-RSL | PK-RSL |
| **[pclx_6_] (mM)** | 32 | 8 |
| **Precipitant** | 1.26 M ammonium sulfate | 1.1 M di-ammonium hydrogen phosphate |
| **Buffer (0.1 M)** | Tris-HCl pH 8.5 | sodium acetate pH 4.5 |
| **Additive (0.2 M)** | lithium sulfate | - |
| **Data Collection***^a^* | | |
| **Light Source** | SOLEIL, PROXIMA-2A | |
| **Wavelength (Å)** | 0.98011 | |
| **Space group** | *I*23 | |
| **Cell constants (Å, °)** | 87.973, 87.973, 87.973  90.0, 90.0, 90.0 | 88.377, 88.377, 88.377  90.0, 90.0, 90.0 |
| **Resolution (Å)** | 35.91-1.19 (1.21-1.19) | 31.25-1.28 (1.30-1.28) |
| **# reflections** | 1158652 (30678) | 1167626 (50411) |
| **# unique reflections** | 36569 (1728) | 29915 (1478) |
| **Multiplicity** | 31.7 (17.8) | 39.0 (34.1) |
| **I/σ (I)** | 25.4 (0.9) | 37.6 (2.3) |
| **Completeness (%)** | 99.7 (94.8) | 100.0 (100.0) |
| ***R*_meas_*^b^* (%)** | 5.8 (264.7) | 4.9 (157.9) |
| ***R*_pim_*^c^* (%)** | 1.0 (60.3) | 0.8 (26.9) |
| **CC_1/2_** | 100.0 (35.1) | 100.0 (82.7) |
| **Solvent content (%)** | 53 | 53 |
| **Refinement** | | |
| ***R*_work_** | 0.183 | 0.195 |
| ***R*_free_** | 0.201 | 0.204 |
| **rmsd bonds (Å)** | 0.004 | 0.005 |
| **rmsd angles (°)** | 0.807 | 0.811 |
| **# molecules in asymmetric unit** | | |
| **Protein chains** | 1 | 1 |
| **pclx_6_** | 1 | 1 |
| **water** | 124 | 120 |
| **Ave. B-factor (Å^2^)** | 23.06 | 27.61 |
| **Clashscore** | 0.68 | 0 |
| **Ramachandran analysis,*^d^* % residues in** | | |
| **favoured regions** | 96.63 | 96.63 |
| **allowed regions** | 3.37 | 3.37 |
| **PDB code** | 9hbf | 9hbg |

*^a^*Values in parentheses correspond to the highest resolution shell; *^b^R*_meas_ = ∑*_hkl_* √(*n/n-1*)∑_i_ |*I_i_*(*hkl*) *-* 〈*I*(*hkl*)〉|/∑*_hkl_* ∑*_i_I_i_*(*hkl*); *^c^R*_pim_ = ∑*_hkl_* √(1/n-1)∑^n^ *_i=1_* |*Ii*(*hkl*) - 〈*I*(*hkl*)〉|/∑*_hkl_* ∑*_i_I_i_*(*hkl*); *^d^*Calculated in MolProbity


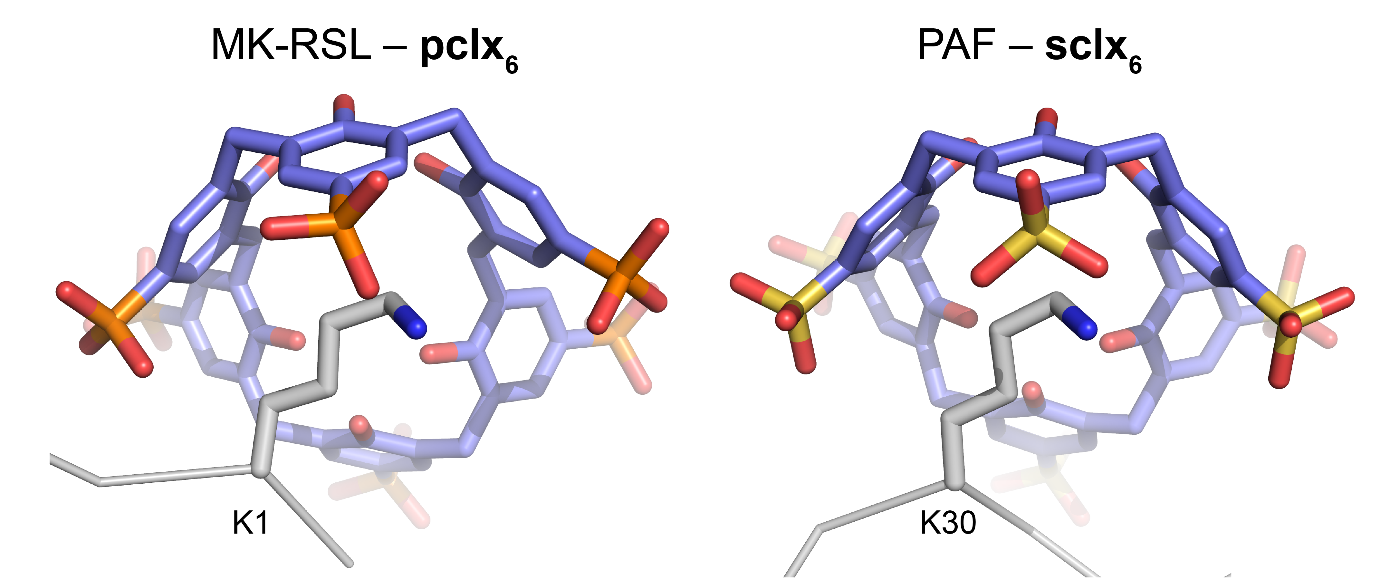


**Figure S10.** The lysine – calix[6]arene synthon in cocrystal structures of MK-RSL – **pclx_6_** (PDB 9hbf) and *Penicillium* antifungal protein – **sclx_6_** (PDB 6hah).^[20]^


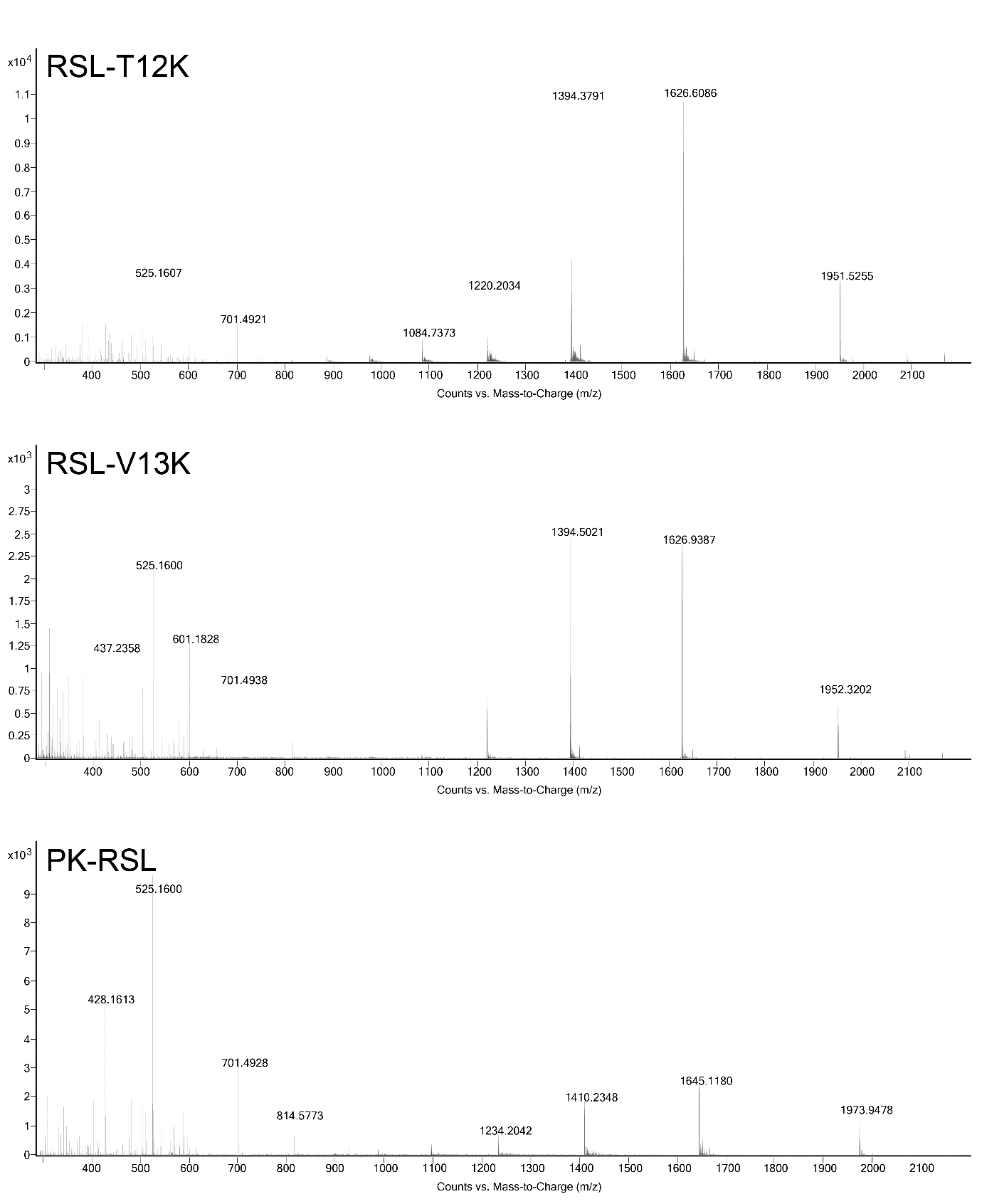


**Figure S11.** ESI+ mass spectrum for PK-RSL.

**Table S3.** Predicted and measured masses from ESI+ mass spectrum.

| **PK-RSL monomer** | | | |
| --- | --- | --- | --- |
| **m/z** | **charge** | **MWt (Da)** | **Error (Da)** |
| 1410.23 | 7+ | 9864.59 | -0.06 |
| 1645.19 | 6+ | 9864.66 | 0.01 |
| 1973.95 | 5+ | 9864.70 | 0.05 |
| Predicted MW (Da)  Deconvoluted MW (Da)  Standard deviation (Da) | | 9864.83 |  |
|  |  | 9864.65 |  |
|  |  | 0.06 |  |


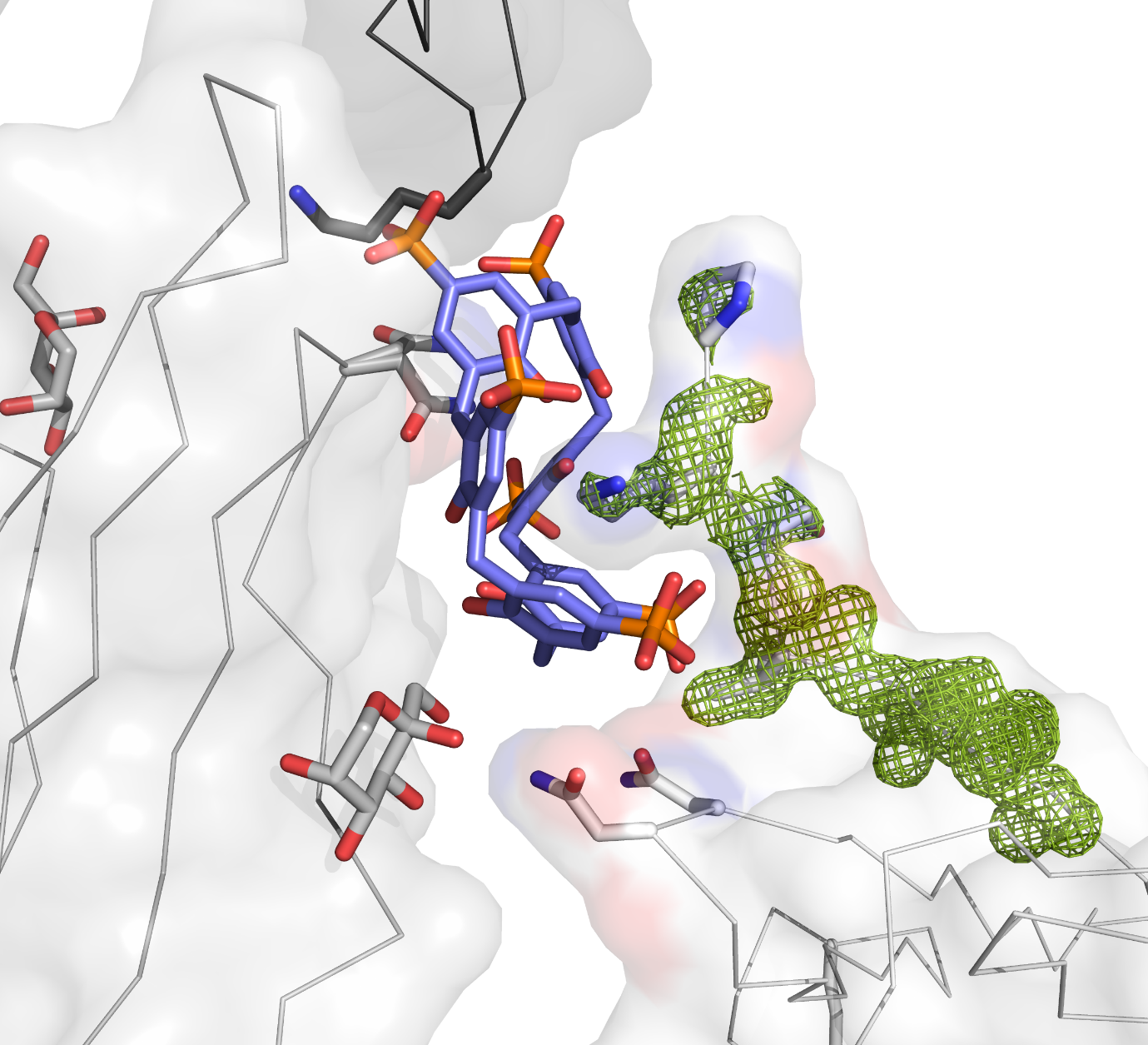


**Figure S12.** The PK-RSL – **pclx_6_** binding mode. This structure is isomorphous to the MK-RSL – **pclx_6_** cocrystal structure (space group *I*23).


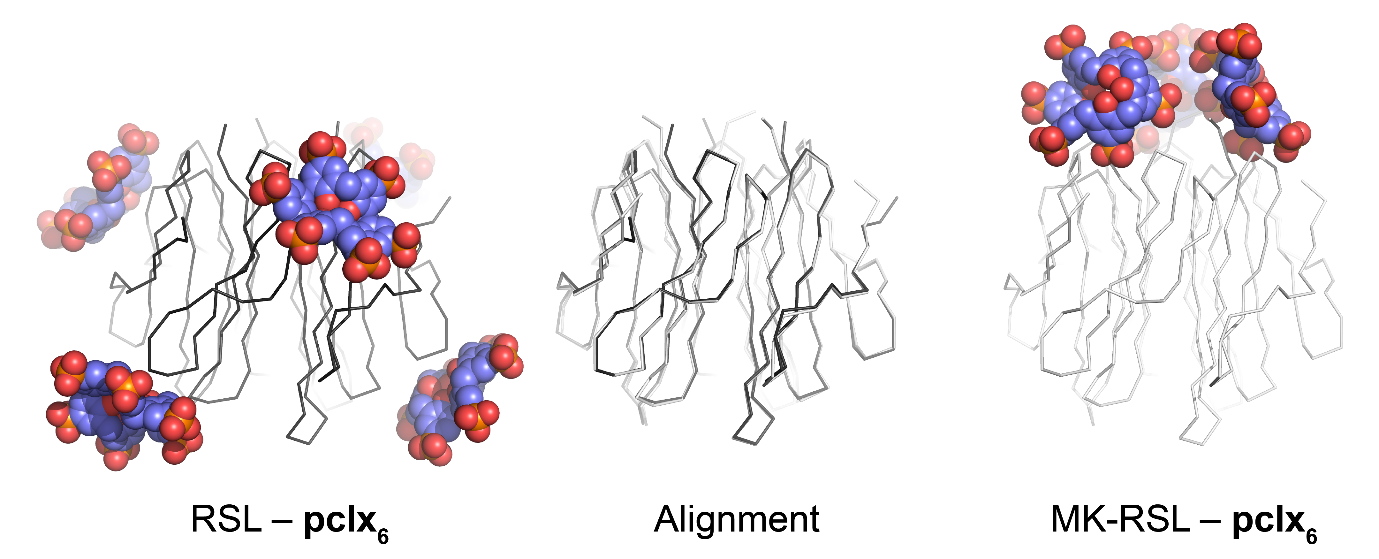


**Figure S13.** Structural alignment of RSL (PDB 9hbd, dark grey) and MK-RSL (PDB 9hbf, light grey). The differences in the protein backbones are minimal.


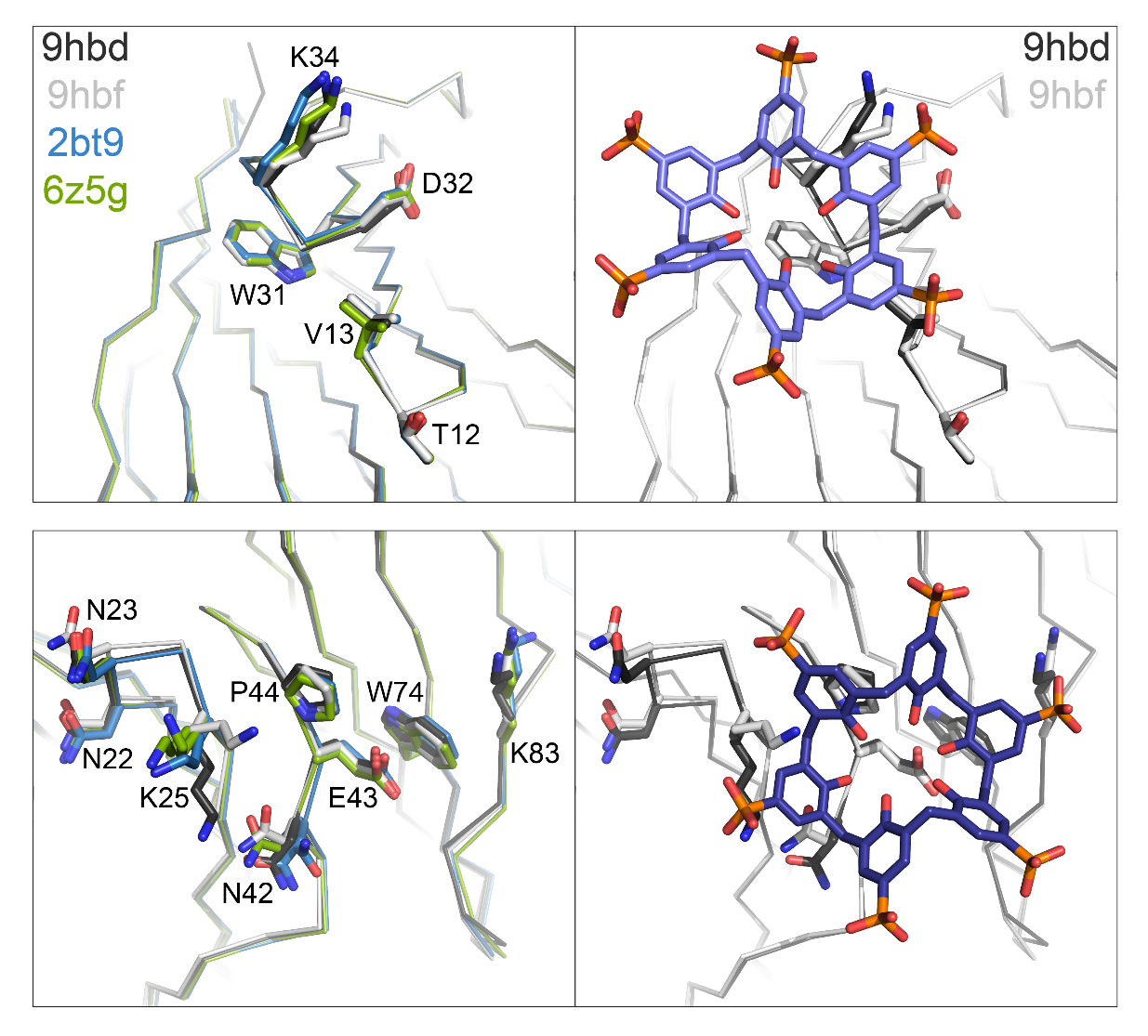


**Figure S14.** Structural superposition of RSL from different structures (PDBs 9hbd, 2bt9, 6z5g) and MK-RSL (PDB 9hbf), focusing on the RSL – **pclx_6_** binding sites. Variations in side-chain conformations (*e.g.* Lys25) are due to calixarene binding.

**
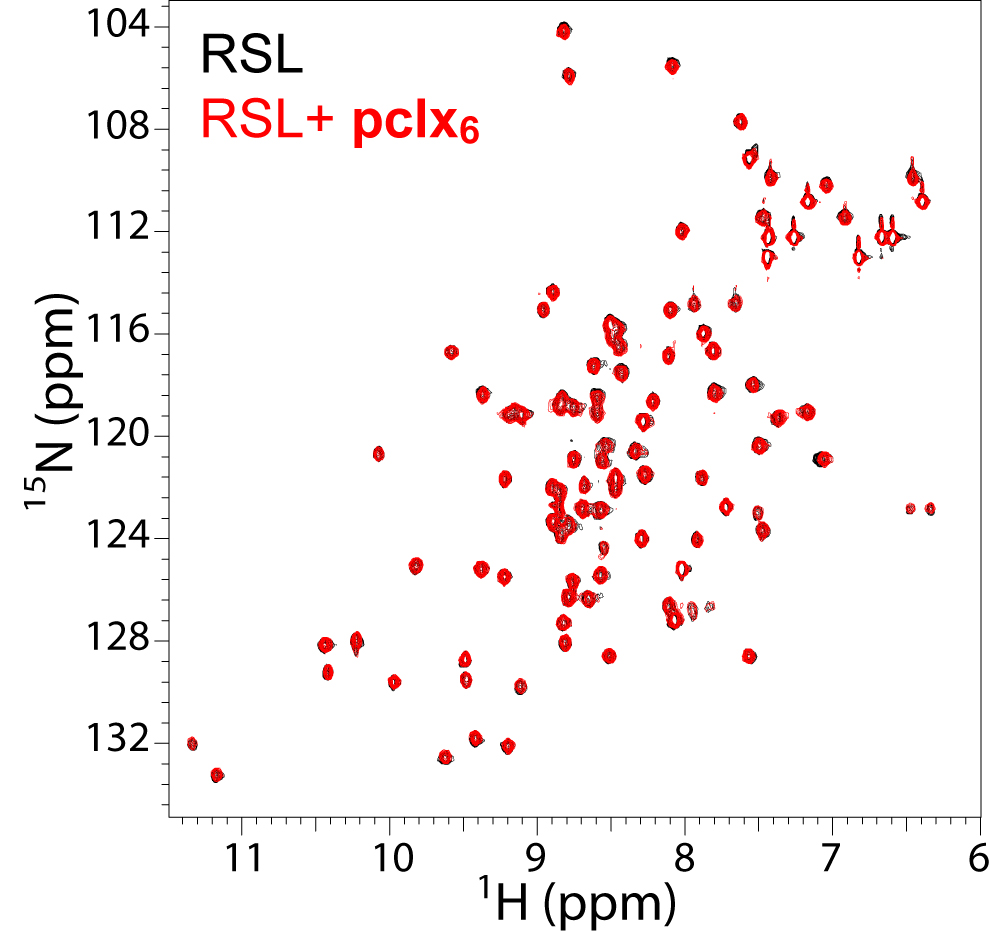
**

**Figure S15.** Superposed **^1^**H−^15^N HSQC spectra (at 30 °C) of 0.1 mM ^15^N-labelled RSL in the absence (black contours) or presence (red) of 0.3 mM **pclx_6_**, in 20 mM potassium phosphate, 50 mM NaCl, 5 mM D-fructose, 10% D_2_O, pH 6.0.

**References**

1. T. E. Clark, M. Makha, A. N. Sobolev, D. Su, H. Rohrs, M. L. Gross, J. L. Atwood, C. L. Raston, Self-Organised Nano-Arrays of *p*-Phosphonic Acid Functionalised Higher Order Calixarenes. *New J. Chem.* **2008**, *32*, 1478–1483.
2. A. D. Martin, C. L. Raston, Multifunctional *p*-Phosphonated Calixarenes. *Chem. Commun.* **2011**, *47*, 9764–9772.
3. K. O. Ramberg, S. Engilberge, T. Skorek, P. B. Crowley, Facile Fabrication of Protein-Macrocycle Frameworks. *J. Am. Chem. Soc.* **2021**, *143*, 1896–1907.
4. K. O. Ramberg, S. Engilberge, F. Guagnini, P. B. Crowley. Protein Recognition by Cucurbit[6]uril: High Affinity N-Terminal Complexation. *Org. Biomol. Chem.* **2021**, *19*, 837–844.
5. R. E. McGovern, H. Fernandes, A. R. Khan, N. P. Power, P. B. Crowley, Protein Camouflage in Cytochrome *c*–Calixarene Complexes. *Nat. Chem.* **2012**, *4*, 527–533.
6. N. M. Mockler, K. O. Ramberg, P. B. Crowley, Protein – Macrocycle Polymorphism: Crystal Form IV of the RSL – Sulfonato-calix[8]arene Complex. *Acta Crystallogr.* **2023**, *D79*, 624–631.
7. M. L. Rennie, G. C. Fox, J. Pérez,P. B. Crowley, Auto-Regulated Protein Assembly on a Supramolecular Scaffold. *Angew. Chem. Int. Ed.* **2018**, *57*, 13764–13769.
8. R. J. Flood, N. M. Mockler, A. Thureau, M. Malinska, P. B. Crowley, Supramolecular Synthons in Protein-Ligand Frameworks. *Cryst. Growth Des.* **2024**, *24*, 2149–2156.
9. C. Vonrhein, C. Flensburg, P. Keller, A. Sharff, O. Smart, W. Paciorek, T. Womack, G. Bricogne, Data Processing and Analysis with the AutoPROC Toolbox. *Acta Crystallogr*. **2011**, *D67*, 293–302.
10. W. Kabsch, XDS. *Acta Crystallogr.* **2010**, *D66*, 125
11. P. R. Evans, G. N. Murshudov, How Good Are My Data and What is the Resolution? *Acta Crystallogr.* **2013**, *D69*, 1204–1214.
12. P. R. Evans, An Introduction to Data Reduction: Space-Group Determination, Scaling and Intensity Statistics. *Acta Crystallogr.* **2011**, D67, 282−292.
13. A. J. McCoy, R. W. Grosse-Kunstleve, P. D. Adams, M. D. Winn, L. C. Storoni, R. J. Read, Phaser Crystallographic Software. *J. Appl. Crystallogr.* **2007**, 40, 658–674.
14. P. Emsley, K. Cowtan, Coot: Model-Building Tools for Molecular Graphics. *Acta Crystallogr.* **2004**, *D60*, 2126–2132.
15. P. D. Adams, P. V. Afonine, G. Bunkoczi, V. B. Chen, I. W. Davis, N. Echols, J. J. Headd, L. W. Hung, G. J. Kapral, R. W. GrosseKunstleve, A. J. McCoy, N. W. Moriarty, R. Oeffner, R. J. Read, D. C. Richardson, J. S. Richardson, T. C. Terwilliger, P. H Zwart, PHENIX: A Comprehensive Python-Based System for Macromolecular Structure Solution. *Acta Crystallogr.* **2010**, *D66*, 213–221.
16. C. J. Williams, J. J. Headd, N. W. Moriarty, M. G. Prisant, L. L. Videau, L. N. Deis, V. Verma, D. A. Keedy, B. J. Hintze, V. B. Chen, S. Jain, S. M. Lewis, W. B. Arendall, J. Snoeyink, P. D. Adams, S. C. Lovell, J. S. Richardson, D. C. Richardson, MolProbity: More and Better Reference Data for Improved All-Atom Structure Validation. *Protein* *Sci*., **2018**, *27*, 293–315.
17. E. Krissinel, K. Henrick, Inference of Macromolecular Assemblies from Crystalline State. *J. Mol. Biol.* **2007**, *372*, 774–797.
18. D. H. Juers, J. Ruffin, MAP_CHANNELS: A Computation Tool to Aid in the Visualization and Characterization of Solvent Channels in Macromolecular Crystals. *J. Appl. Crystallogr.* **2014**, *47*, 2105–2108.
19. M. L. Rennie, A. M. Doolan, C. L. Raston, P. B. Crowley, Protein Dimerization on a Phosphonated Calix[6]arene Disc. *Angew. Chem. Int. Ed.* **2017**, *56*, 5517–5521.
20. J. M. Alex, M. L. Rennie, S. Engilberge, G. Lehoczki, H. Dorottya, Á. Fizil, G. Batta, P. B. Crowley, Calixarene-Mediated Assembly of a Small Antifungal Protein. *IUCrJ* **2019**, *6*, 238–247.
